# Supplementary figures and images for: SOD1 is a synthetic-lethal target in PPM1D-mutant leukemia cells
Source: eLife. 2024 Jun 18;12:RP91611. doi: 10.7554/eLife.91611 (PMC11186636; doi:10.7554/eLife.91611)

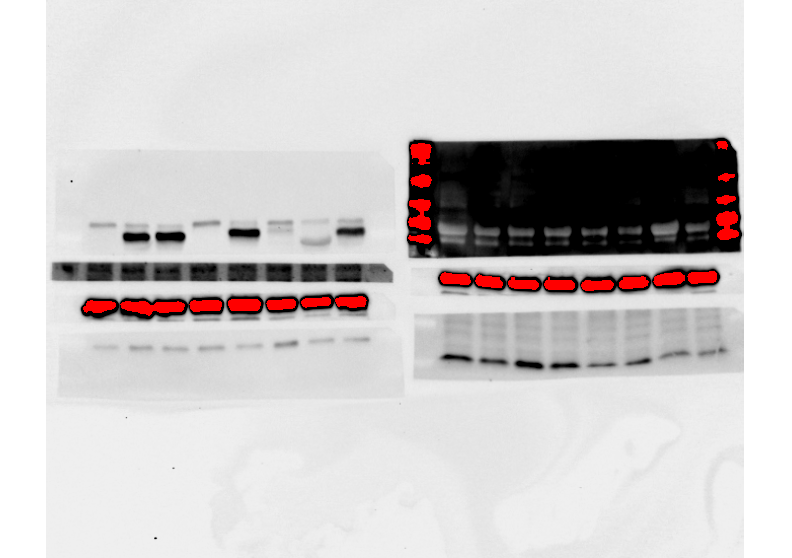

Supplement: Figure 1—figure supplement 1—source data 1. [file elife-91611-fig1-figsupp1-data1.zip › Figure 1-figure supplement 1-source data 1/Sup1A_ZhangL_PPM1D_Raw.tif]

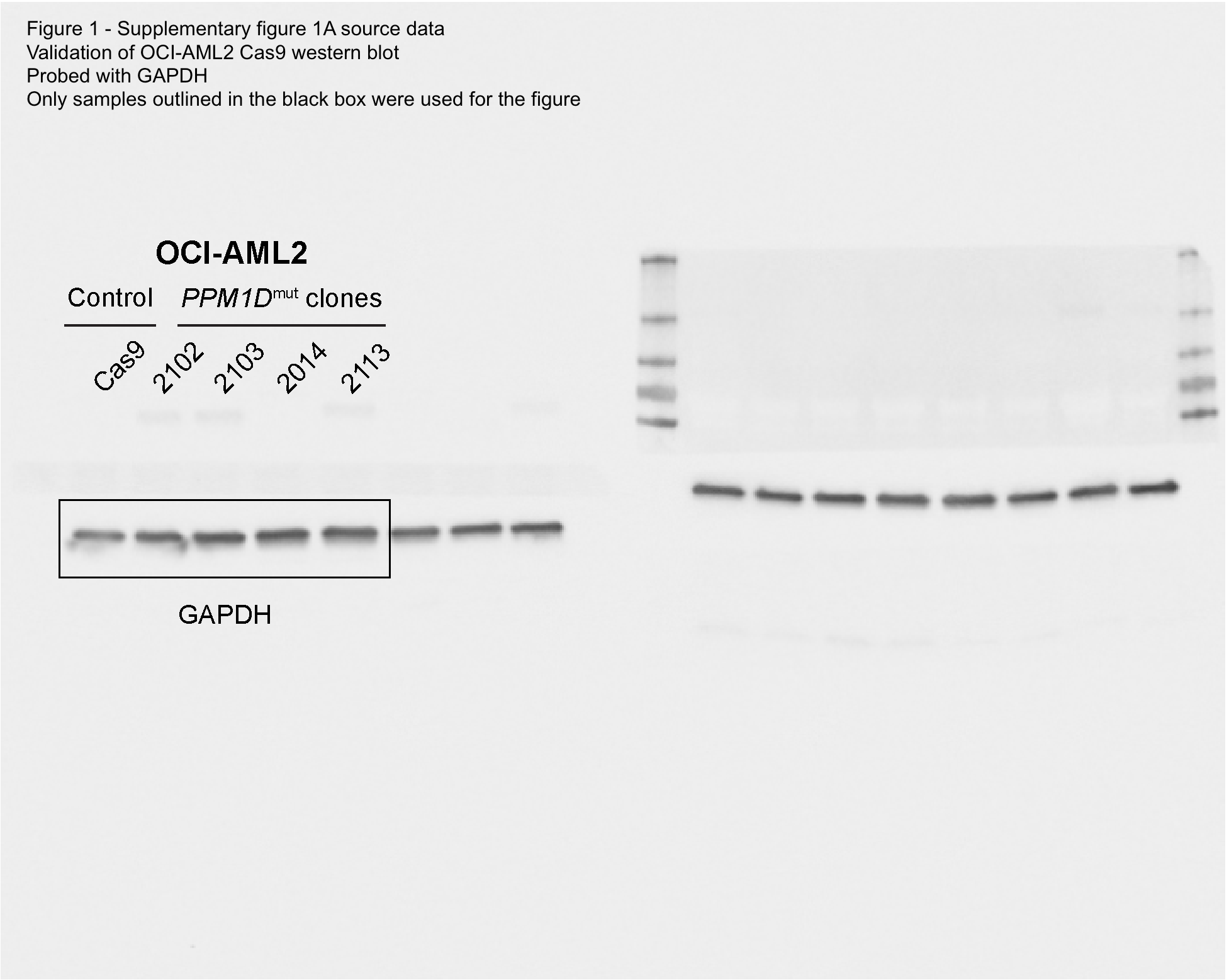

Supplement: Figure 1—figure supplement 1—source data 1. [file elife-91611-fig1-figsupp1-data1.zip › Figure 1-figure supplement 1-source data 1/Sup1A_ZhangL_GAPDH_An.tif]

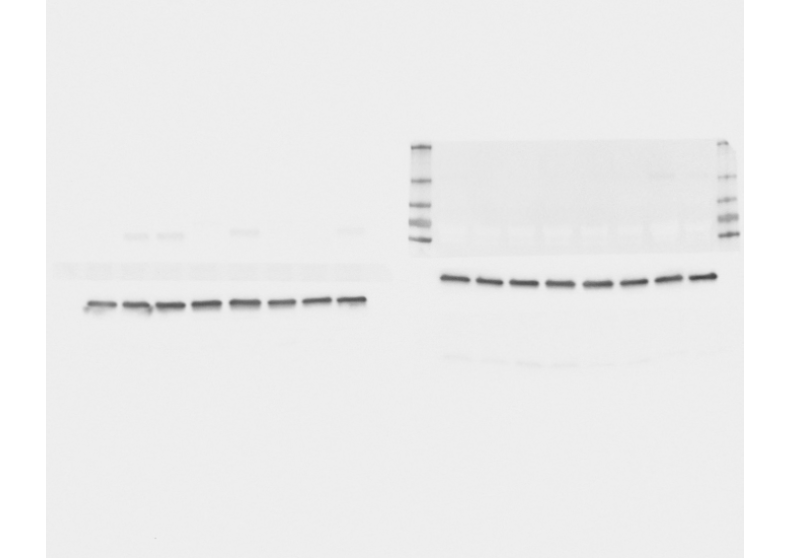

Supplement: Figure 1—figure supplement 1—source data 1. [file elife-91611-fig1-figsupp1-data1.zip › Figure 1-figure supplement 1-source data 1/Sup1A_ZhangL_GAPDH_Raw.tif]

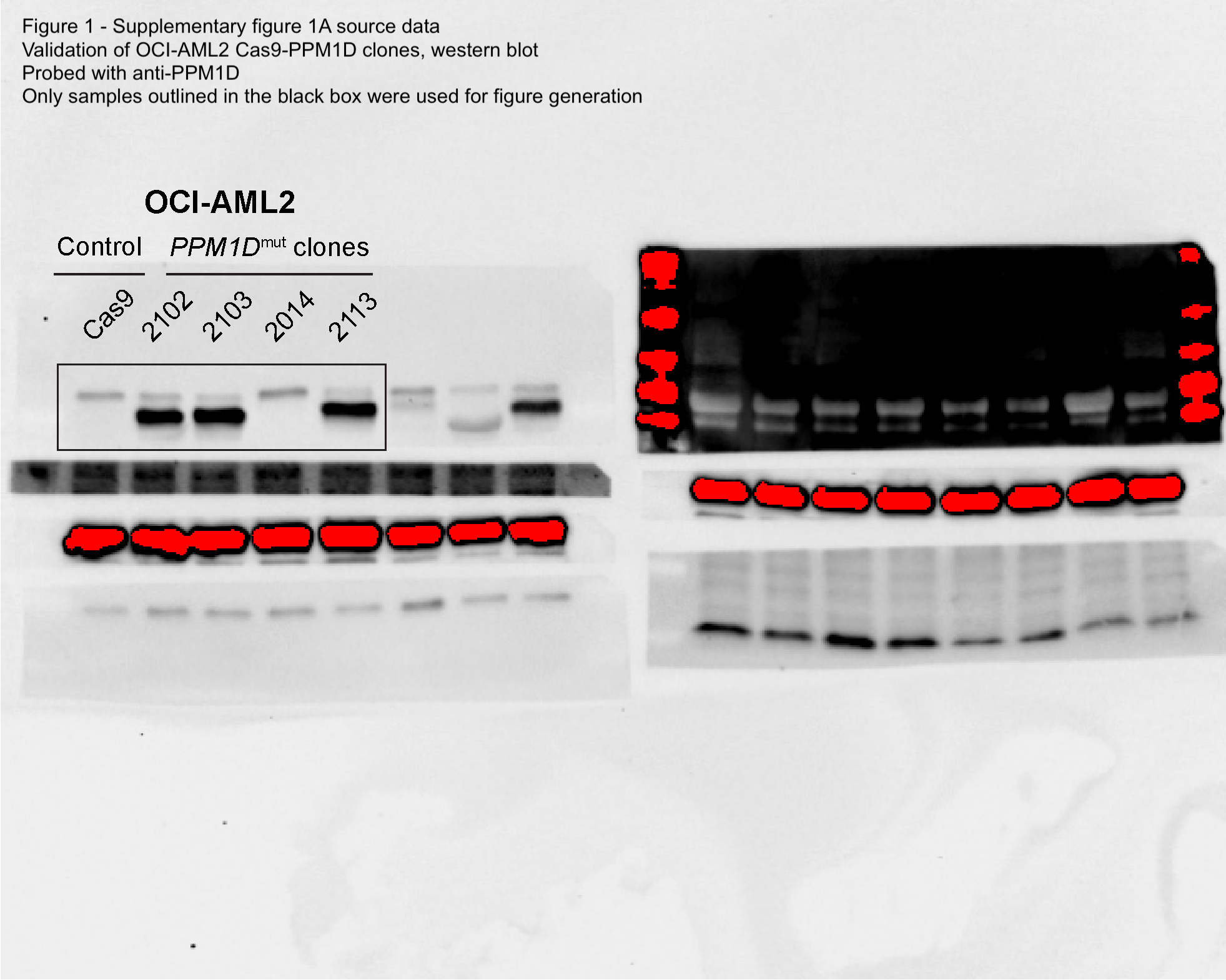

Supplement: Figure 1—figure supplement 1—source data 1. [file elife-91611-fig1-figsupp1-data1.zip › Figure 1-figure supplement 1-source data 1/Sup1A_ZhangL_PPM1D_An.tif]

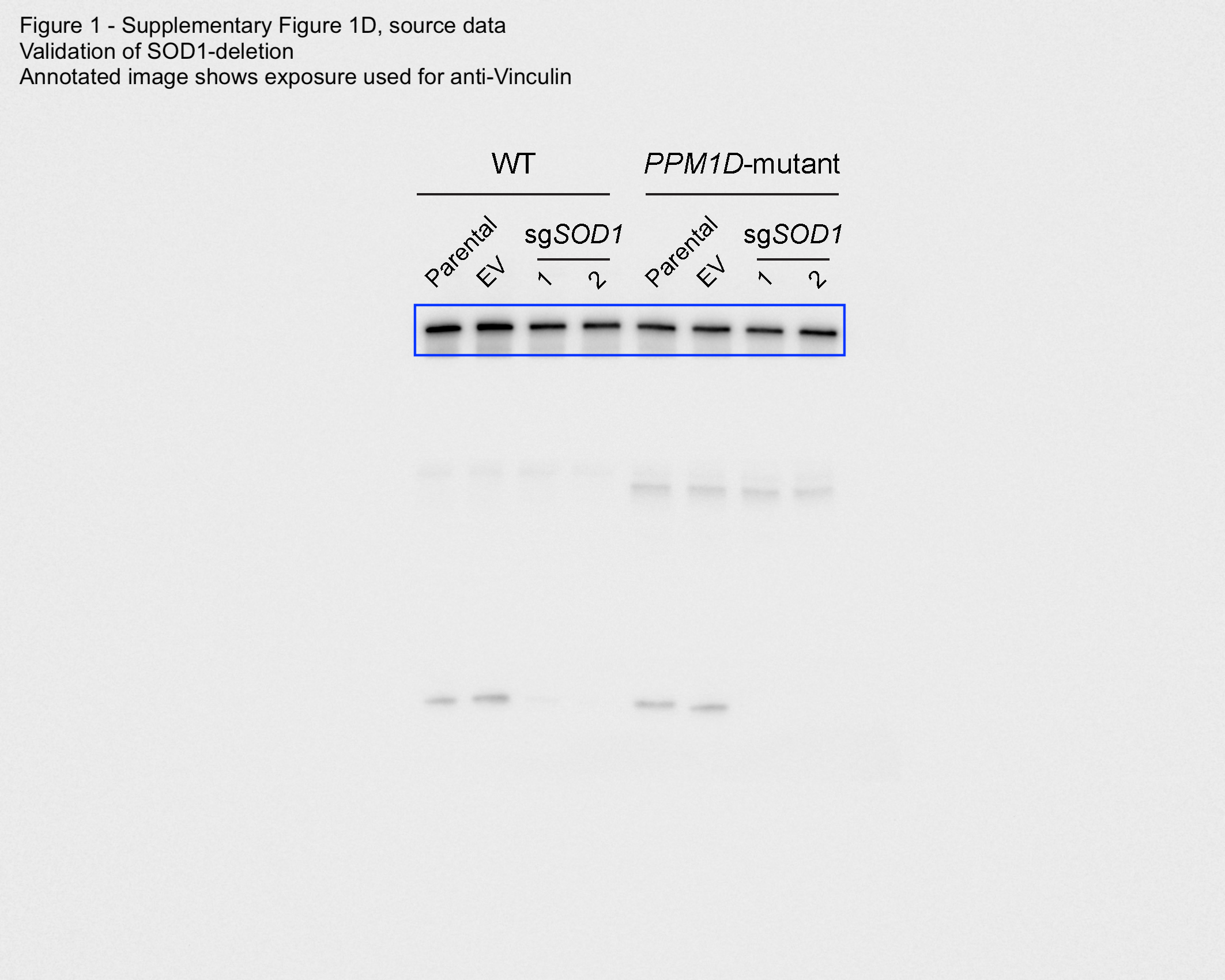

Supplement: Figure 1—figure supplement 1—source data 2. [file elife-91611-fig1-figsupp1-data2.zip › Figure 1-figure supplement 1-source data 2/Supp1D_ZhangL_Vinculin_An.png]

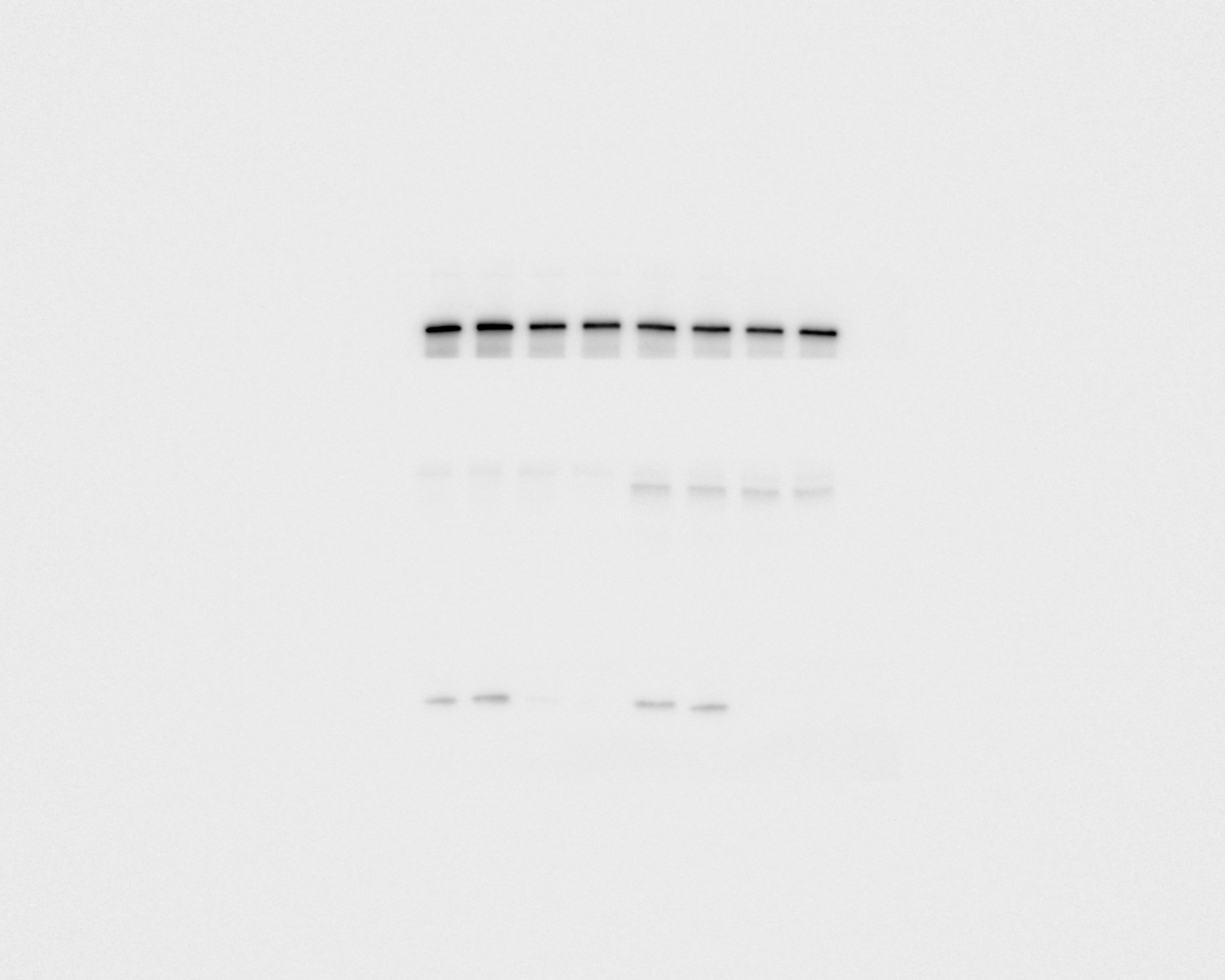

Supplement: Figure 1—figure supplement 1—source data 2. [file elife-91611-fig1-figsupp1-data2.zip › Figure 1-figure supplement 1-source data 2/Supp1D_ZhangL_Vinculin_Raw.png]

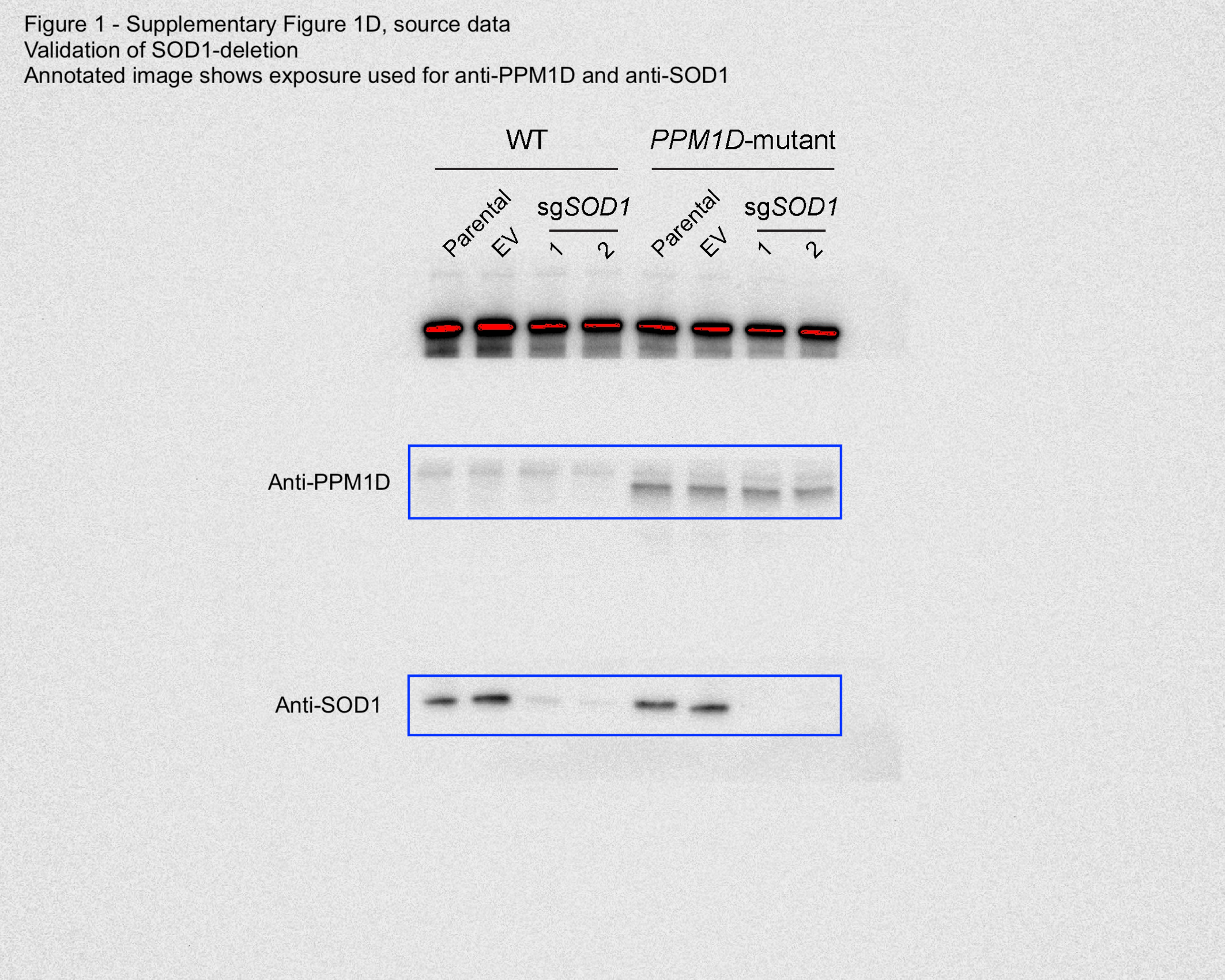

Supplement: Figure 1—figure supplement 1—source data 2. [file elife-91611-fig1-figsupp1-data2.zip › Figure 1-figure supplement 1-source data 2/Supp1D_ZhangL_PPM1D_SOD1_An.png]

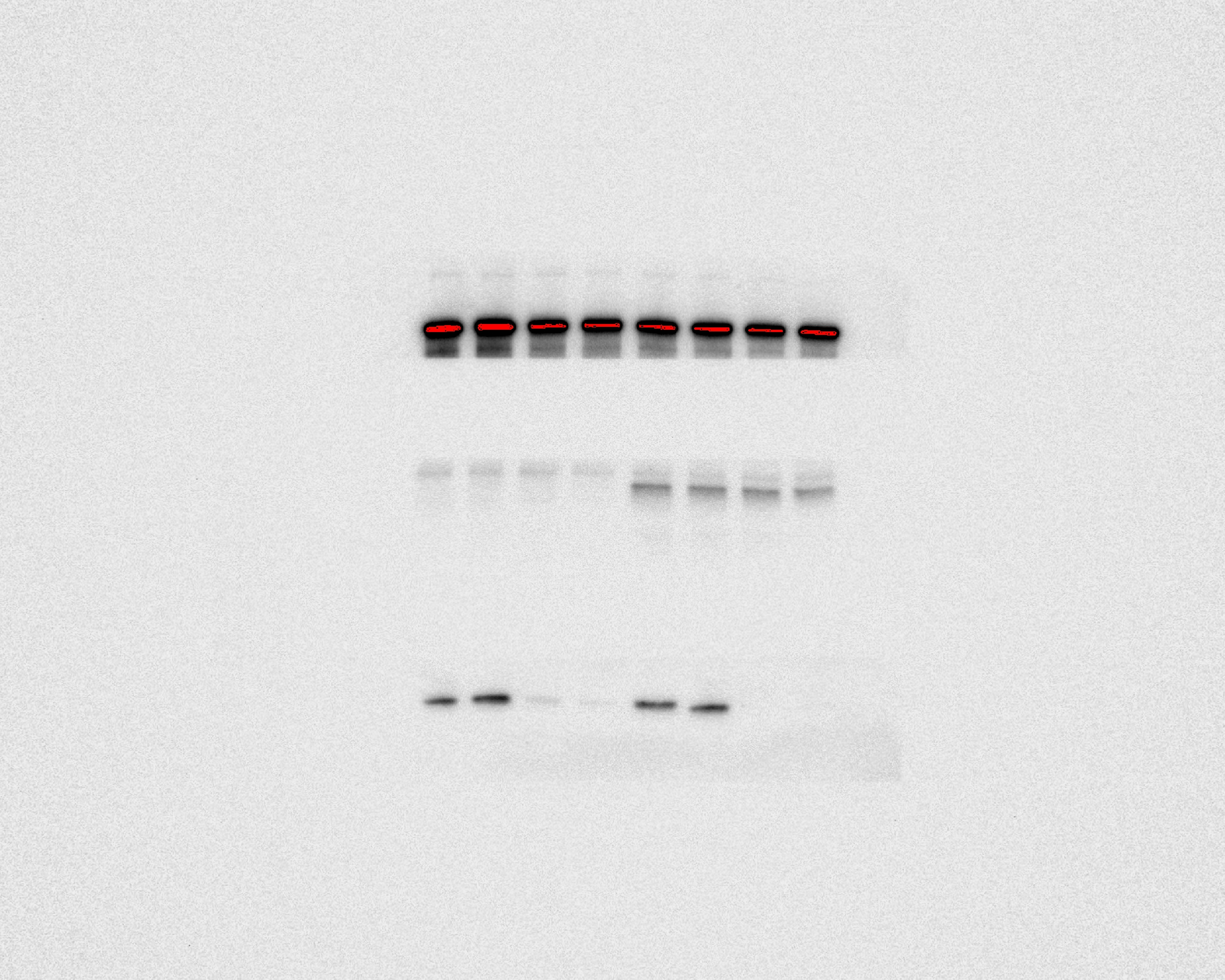

Supplement: Figure 1—figure supplement 1—source data 2. [file elife-91611-fig1-figsupp1-data2.zip › Figure 1-figure supplement 1-source data 2/Supp1D_ZhangL_PPM1D_SOD1_Raw.png]

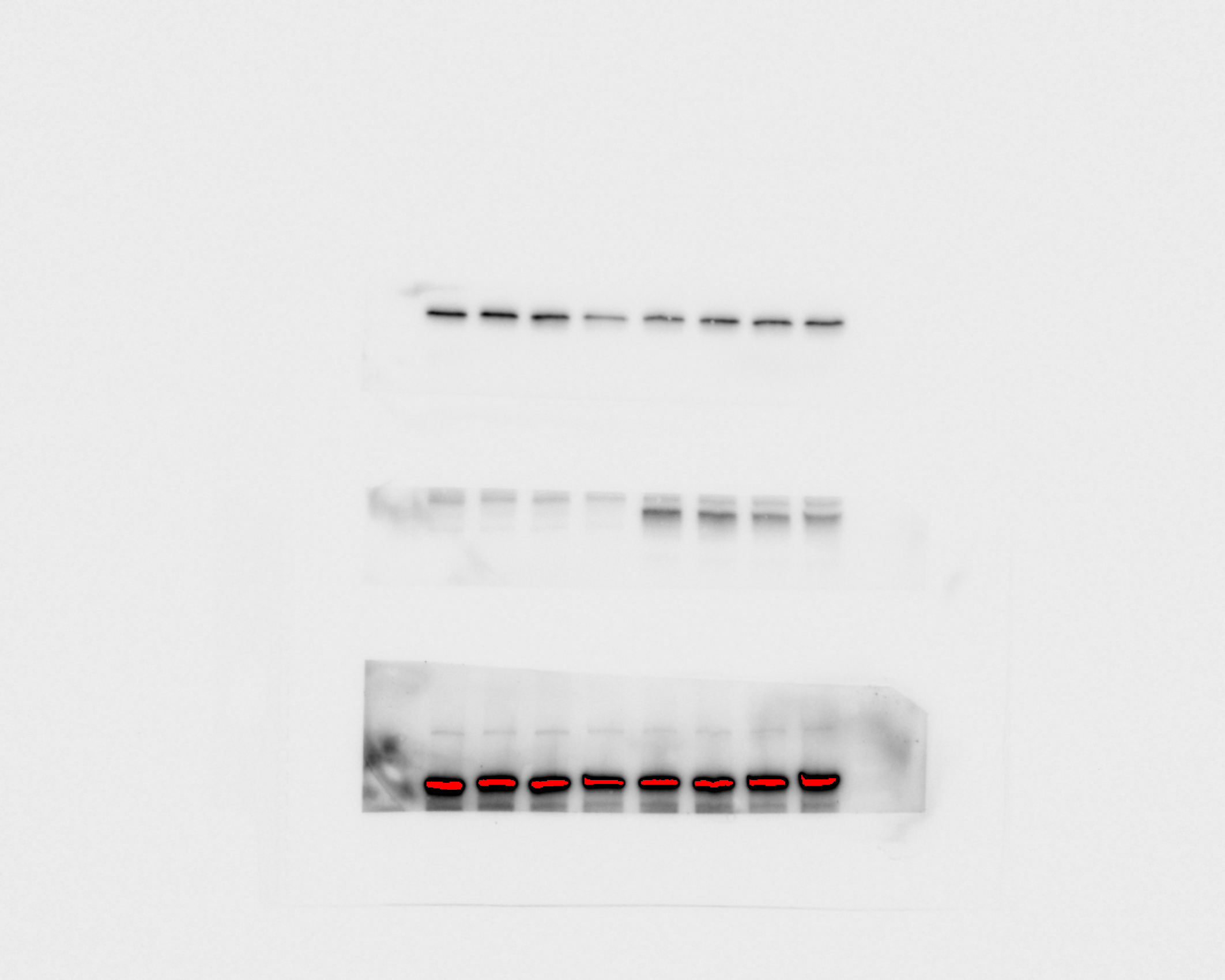

Supplement: Figure 2—figure supplement 2—source data 1. [file elife-91611-fig2-figsupp2-data1.zip › Figure 2-figure supplement 2-source data 1/Fig2Sup2C_ZhangL_SOD2_Raw.png]

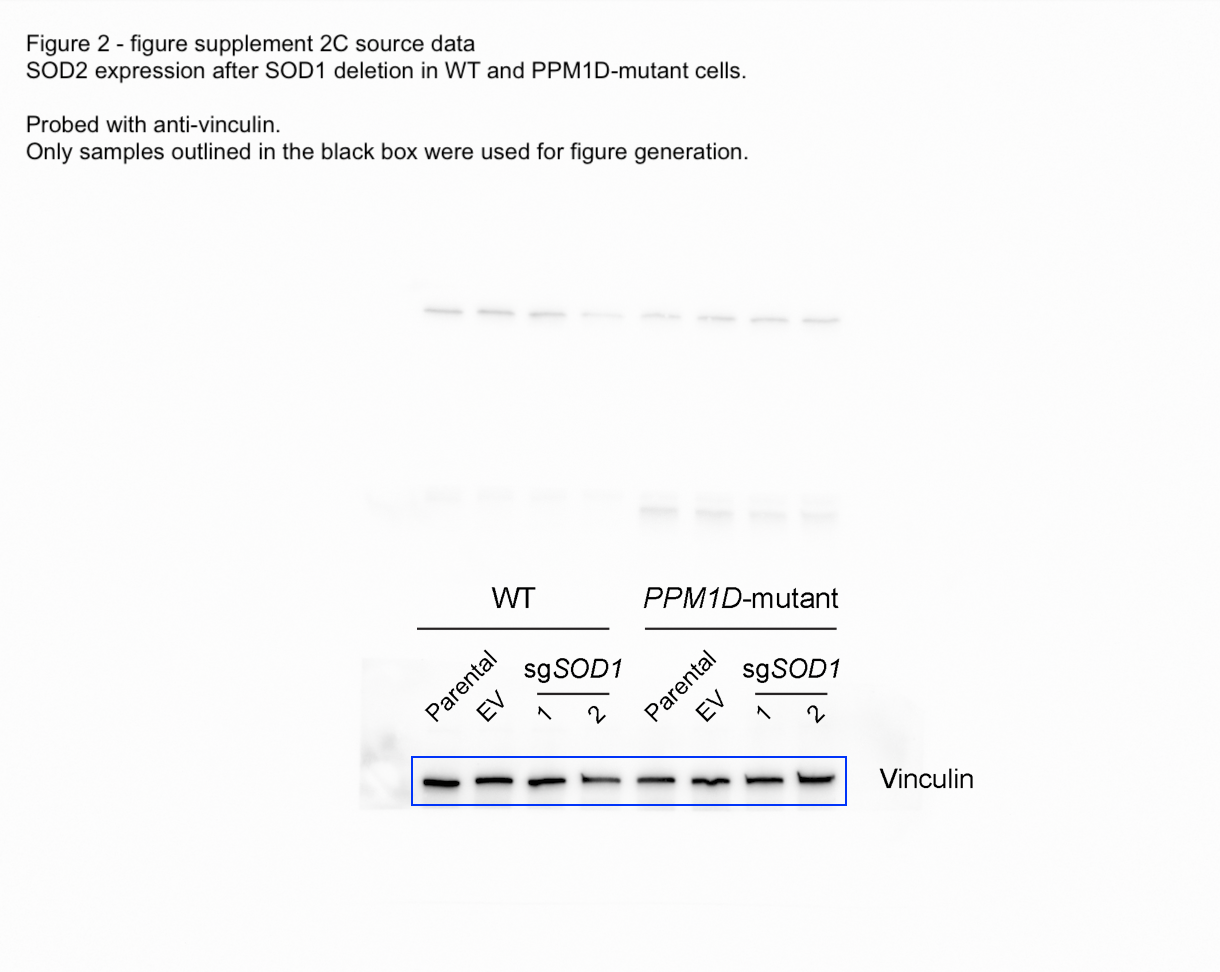

Supplement: Figure 2—figure supplement 2—source data 1. [file elife-91611-fig2-figsupp2-data1.zip › Figure 2-figure supplement 2-source data 1/Fig2Sup2C_ZhangL_Vinculin_An.png]

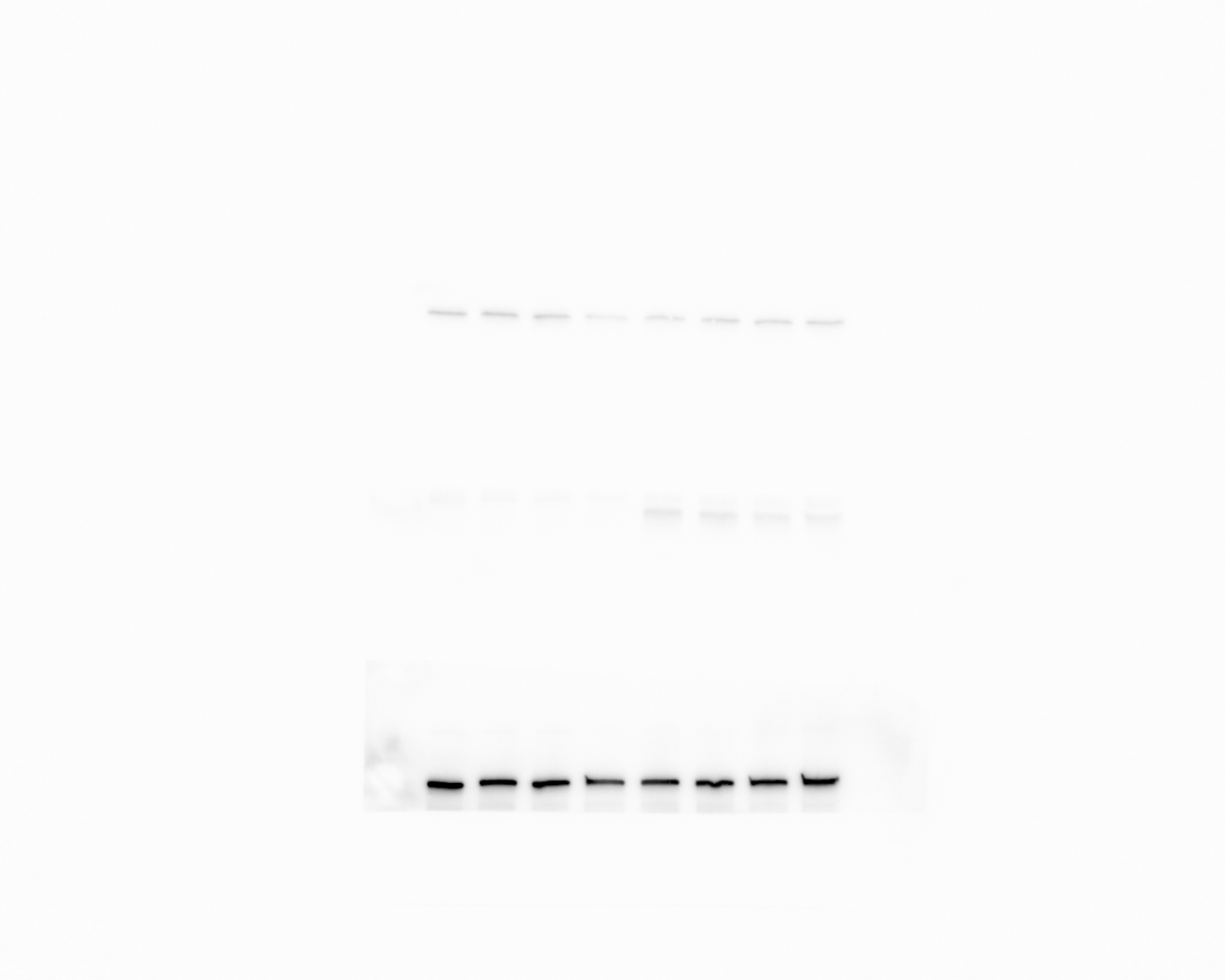

Supplement: Figure 2—figure supplement 2—source data 1. [file elife-91611-fig2-figsupp2-data1.zip › Figure 2-figure supplement 2-source data 1/Fig2Sup2C_ZhangL_Vinculin_Raw.tif]

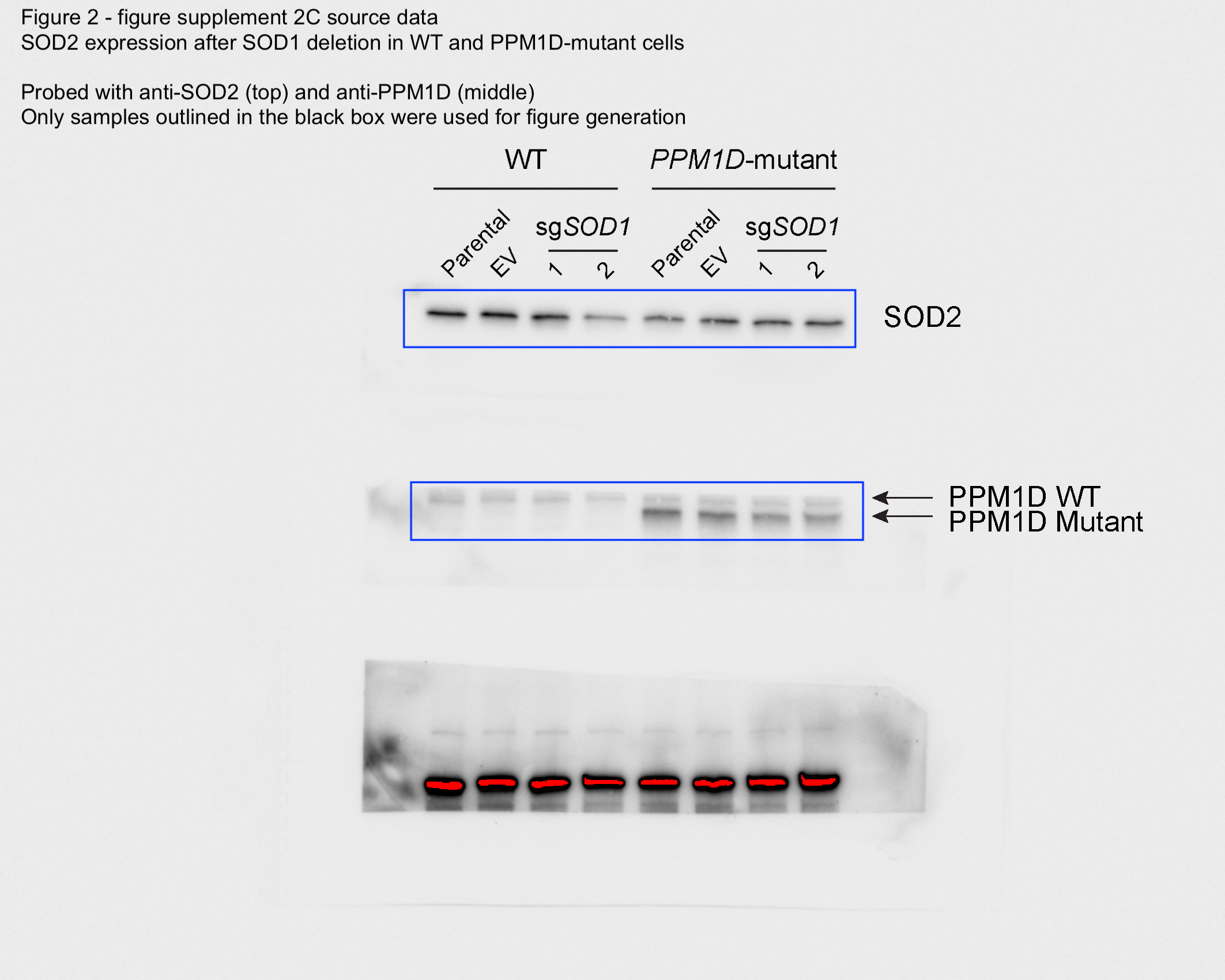

Supplement: Figure 2—figure supplement 2—source data 1. [file elife-91611-fig2-figsupp2-data1.zip › Figure 2-figure supplement 2-source data 1/Fig2Sup2C_ZhangL_SOD2_An.png]

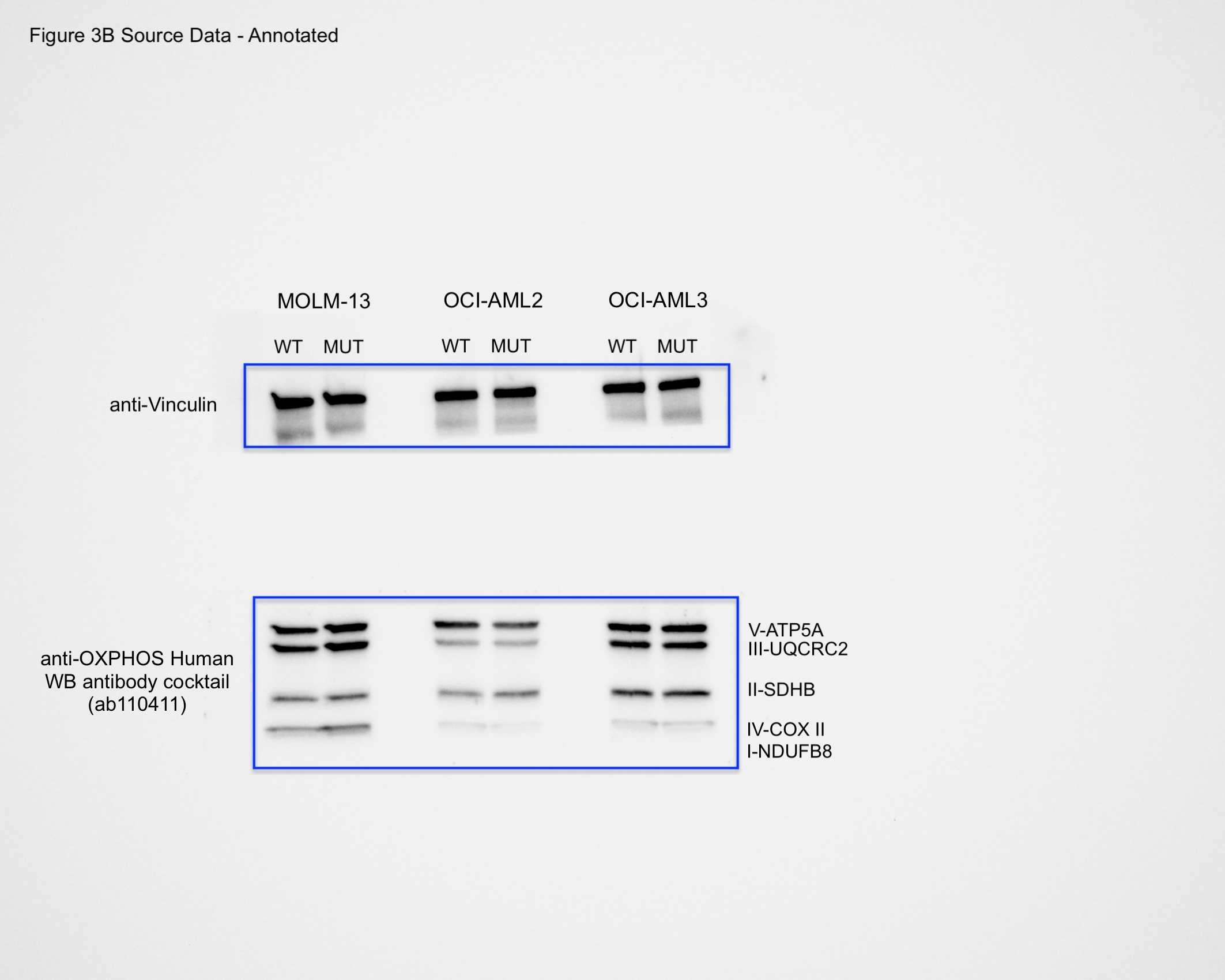

Supplement: Figure 3—source data 1. [file elife-91611-fig3-data1.zip › Figure 3-source data 1/Fig3B_ZhangL_An.jpg]

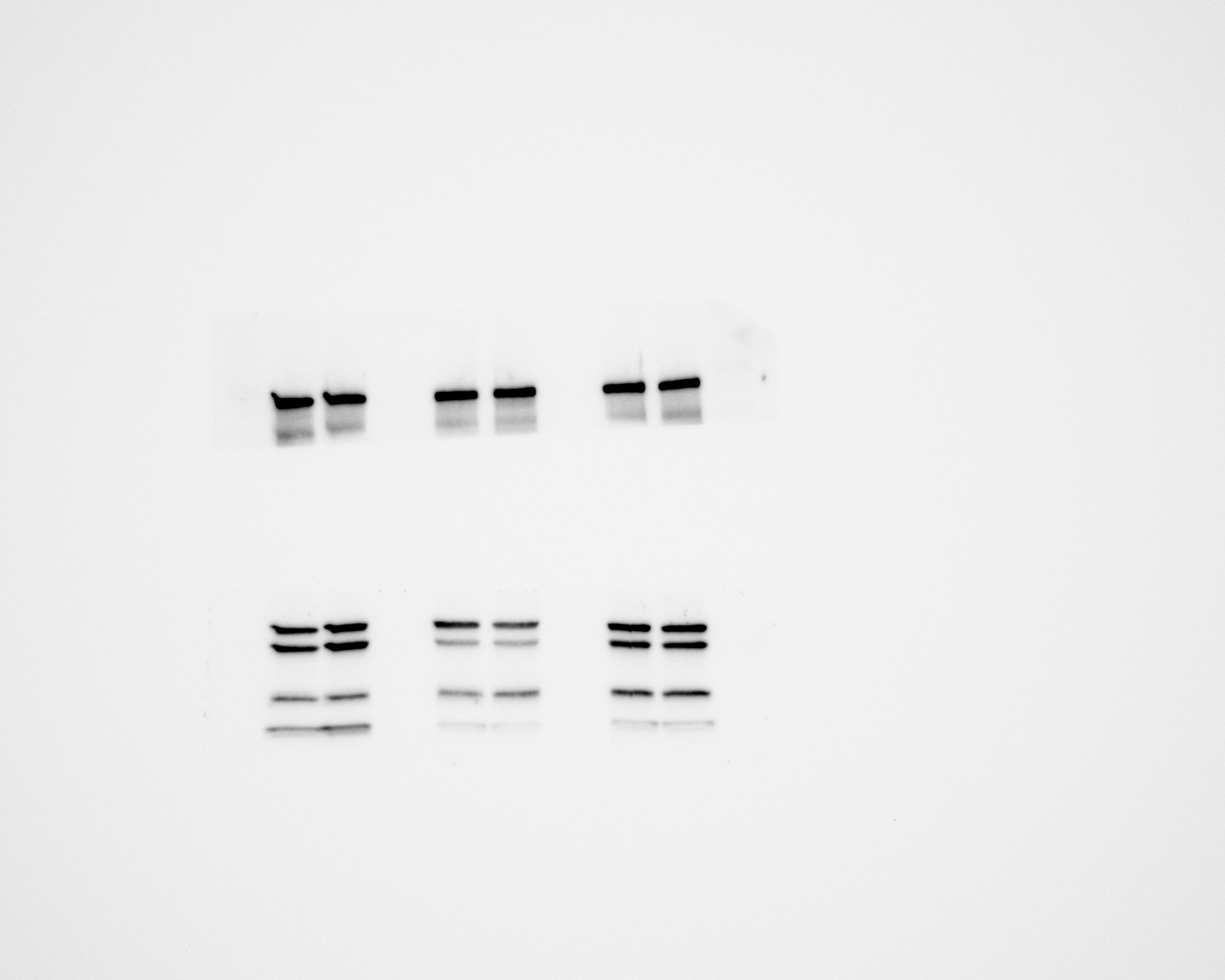

Supplement: Figure 3—source data 1. [file elife-91611-fig3-data1.zip › Figure 3-source data 1/Fig3B_ZhangL_Raw.jpg]

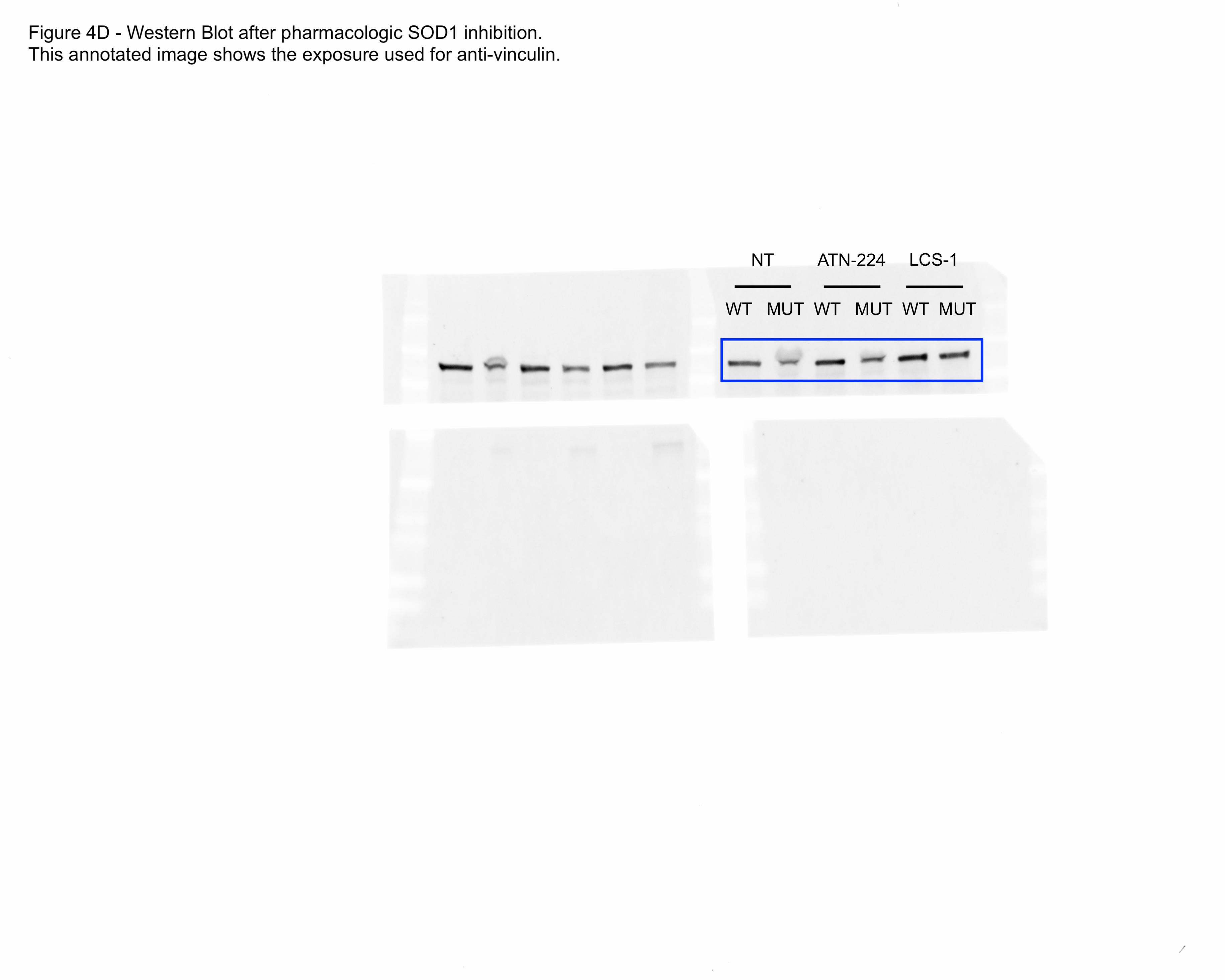

Supplement: Figure 4—source data 4. [file elife-91611-fig4-data4.zip › Figure 4-source data 4/SOD1-inhibition Western Blot (Right) - Annotated/Fig4D_ZhangL_Vinculin_An.png]

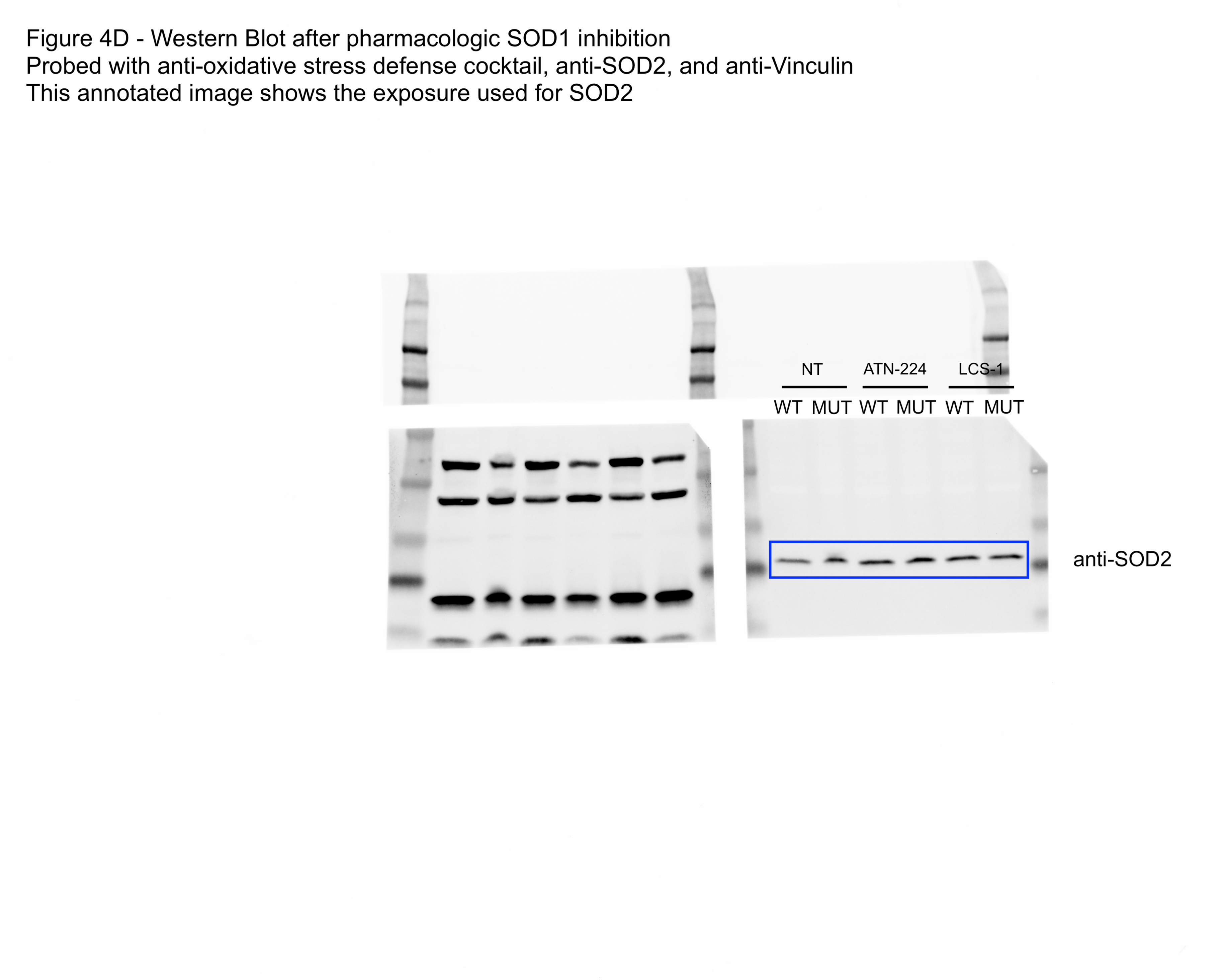

Supplement: Figure 4—source data 4. [file elife-91611-fig4-data4.zip › Figure 4-source data 4/SOD1-inhibition Western Blot (Right) - Annotated/Fig4D_ZhangL_SOD2_An.png]

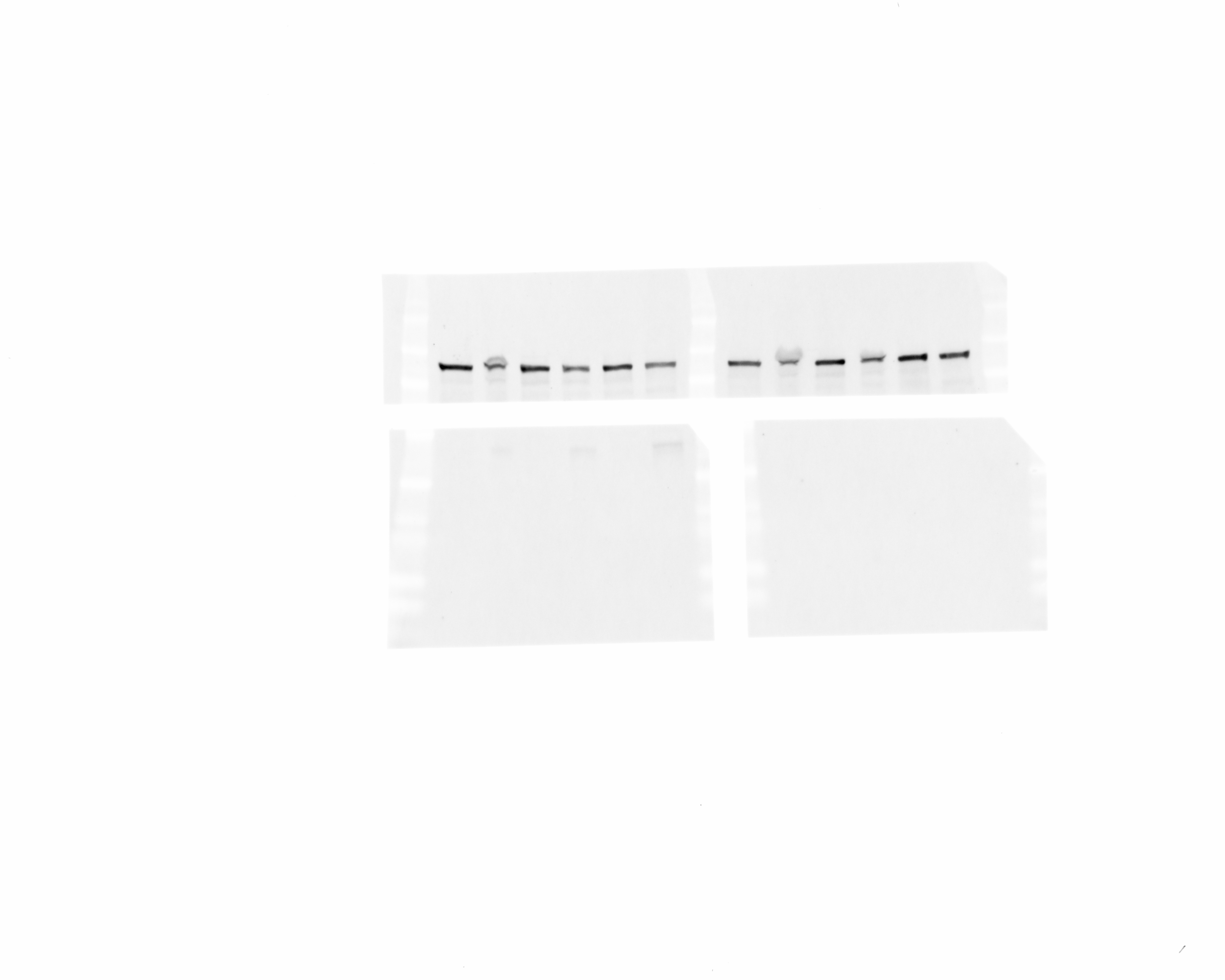

Supplement: Figure 4—source data 4. [file elife-91611-fig4-data4.zip › Figure 4-source data 4/SOD1-inhibition Western Blot (Right) - Annotated/Fig4D_ZhangL_Vinculin_Raw.tif]

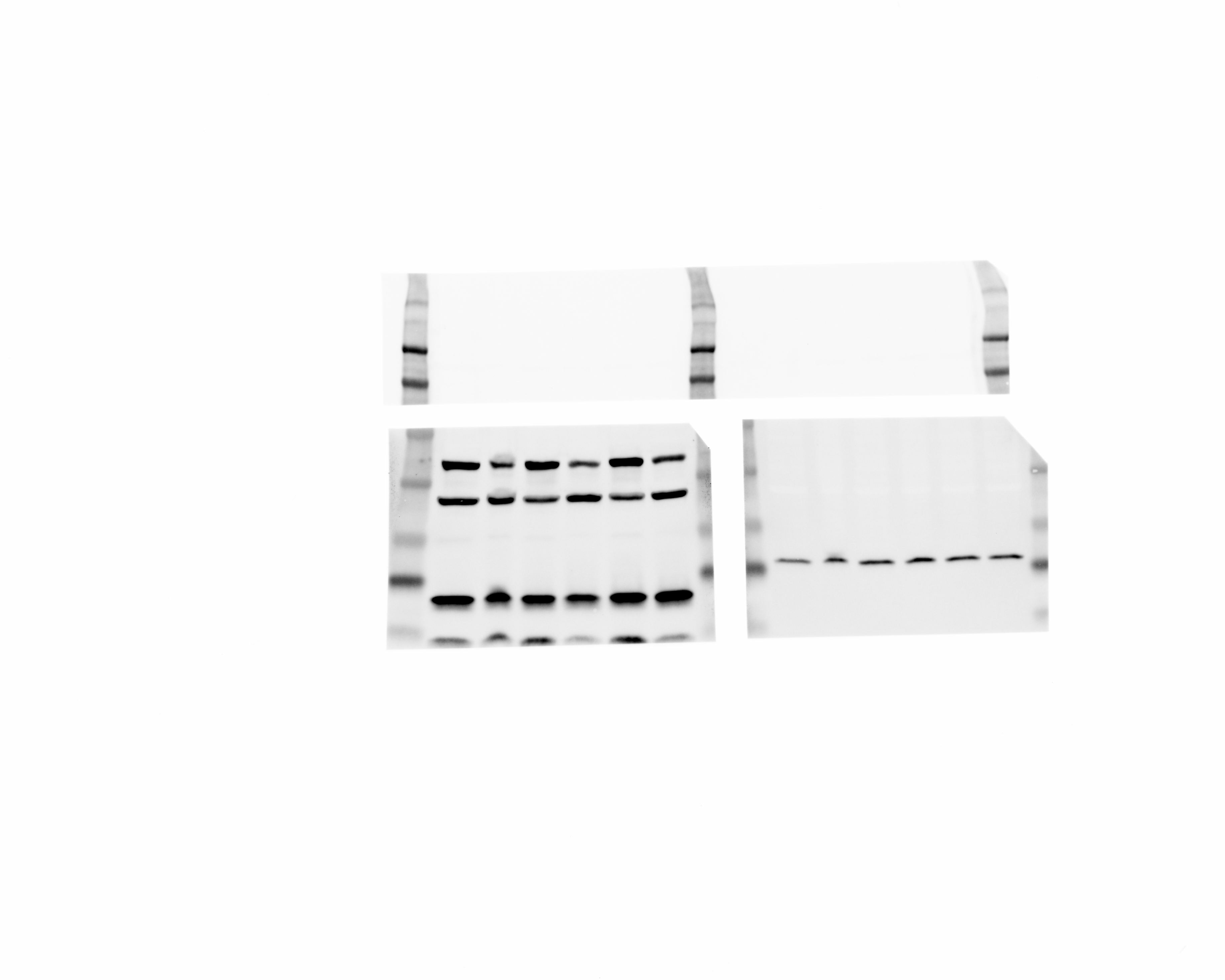

Supplement: Figure 4—source data 4. [file elife-91611-fig4-data4.zip › Figure 4-source data 4/SOD1-inhibition Western Blot (Right) - Annotated/Fig4D_ZhangL_SOD2_Raw.tif]

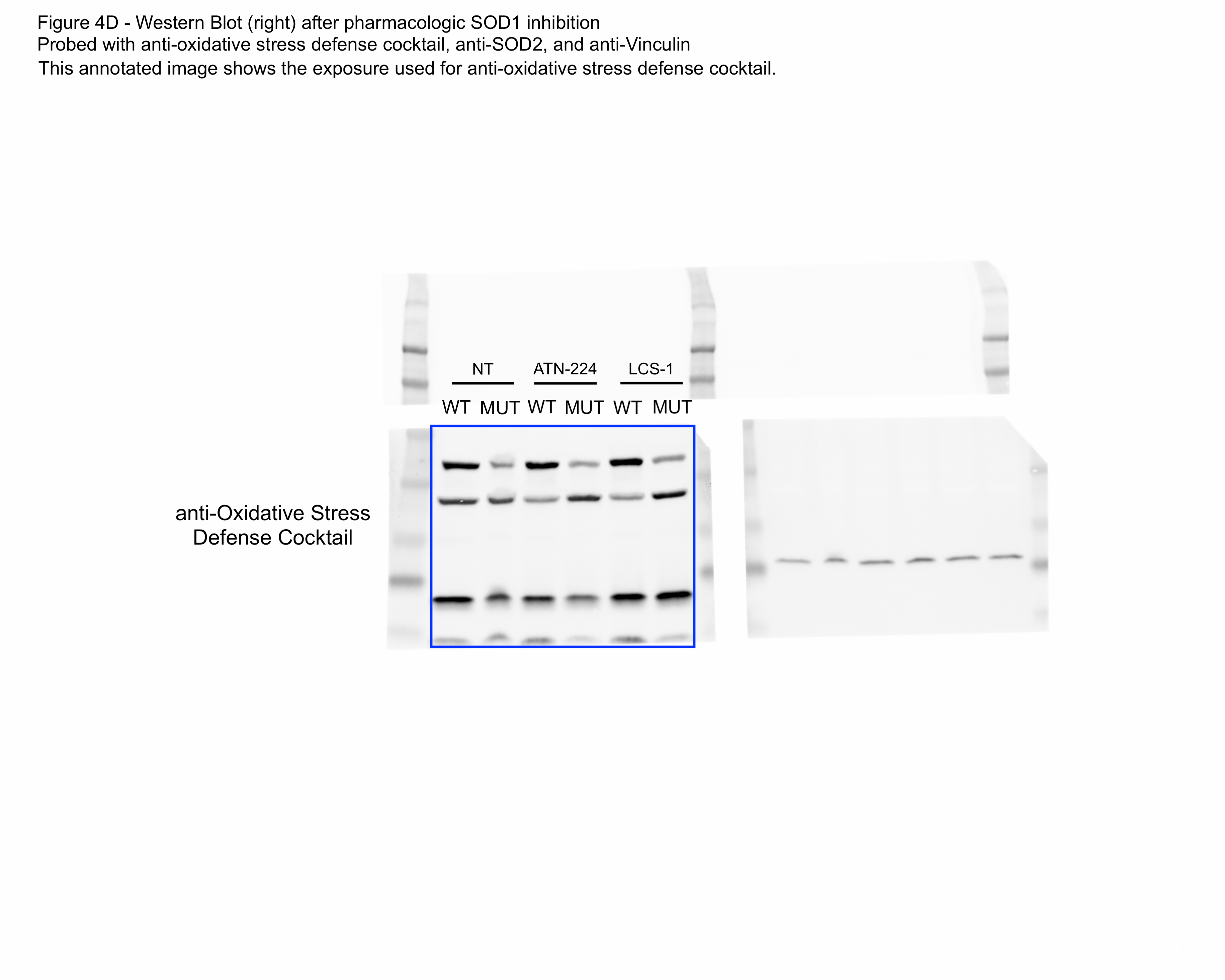

Supplement: Figure 4—source data 4. [file elife-91611-fig4-data4.zip › Figure 4-source data 4/SOD1-inhibition Western Blot (Right) - Annotated/Fig4D_ZhangL_OxStress_An.png]

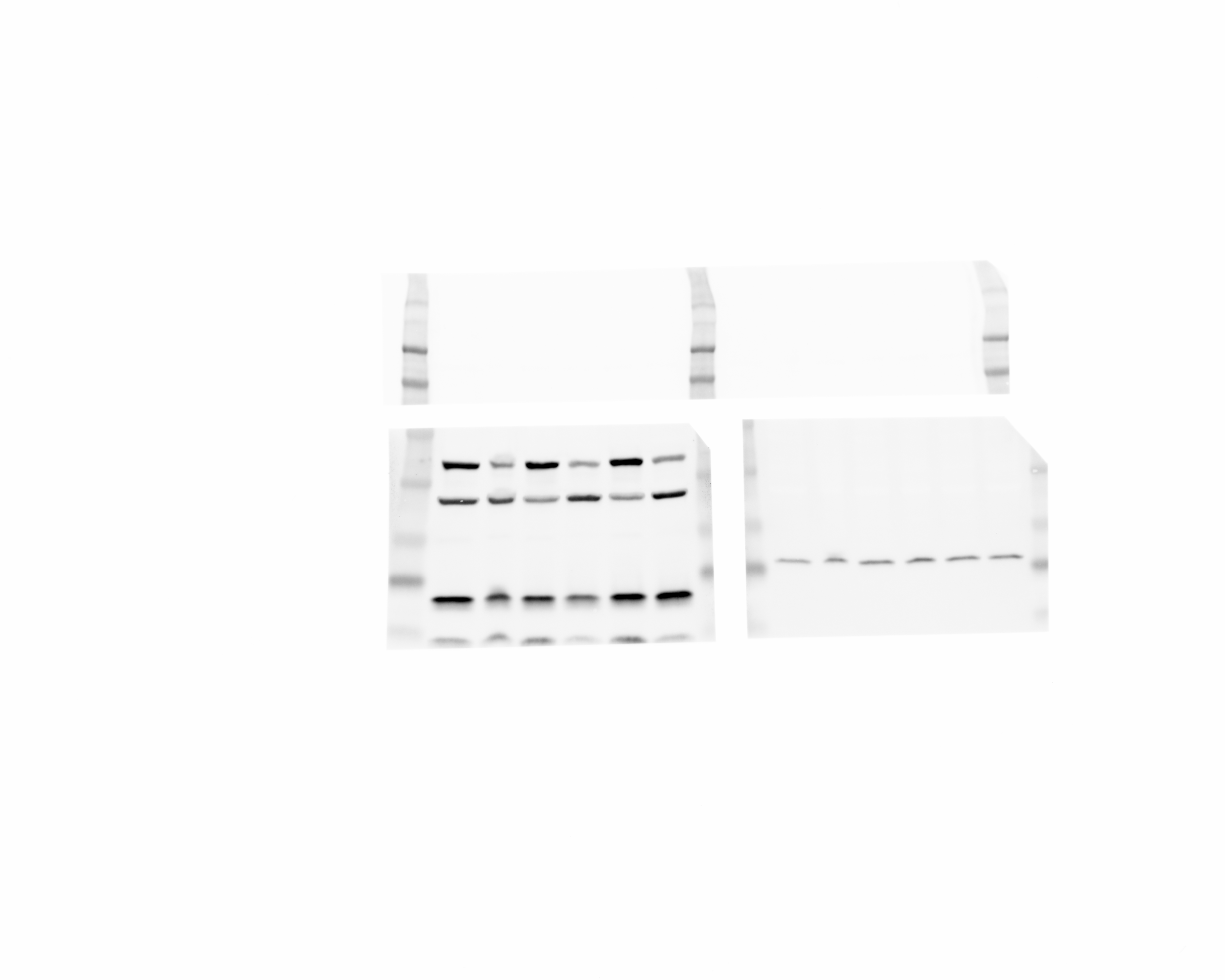

Supplement: Figure 4—source data 4. [file elife-91611-fig4-data4.zip › Figure 4-source data 4/SOD1-inhibition Western Blot (Right) - Annotated/Fig4D_ZhangL_OxStress_Raw.png]

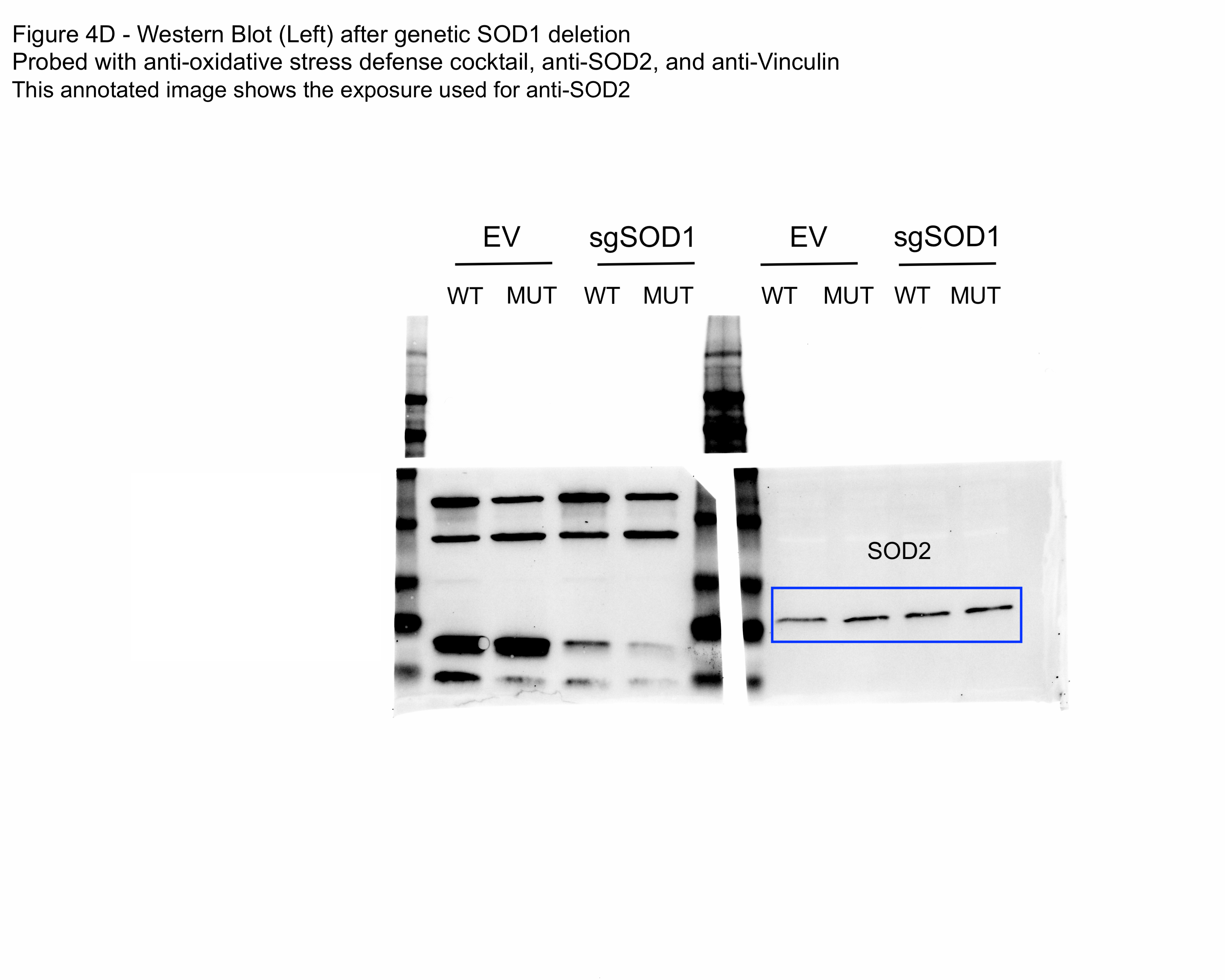

Supplement: Figure 4—source data 4. [file elife-91611-fig4-data4.zip › Figure 4-source data 4/SOD1-KO Western Blot (Left) - Annotated/Fig4D_ZhangL_SOD2_An.tif]

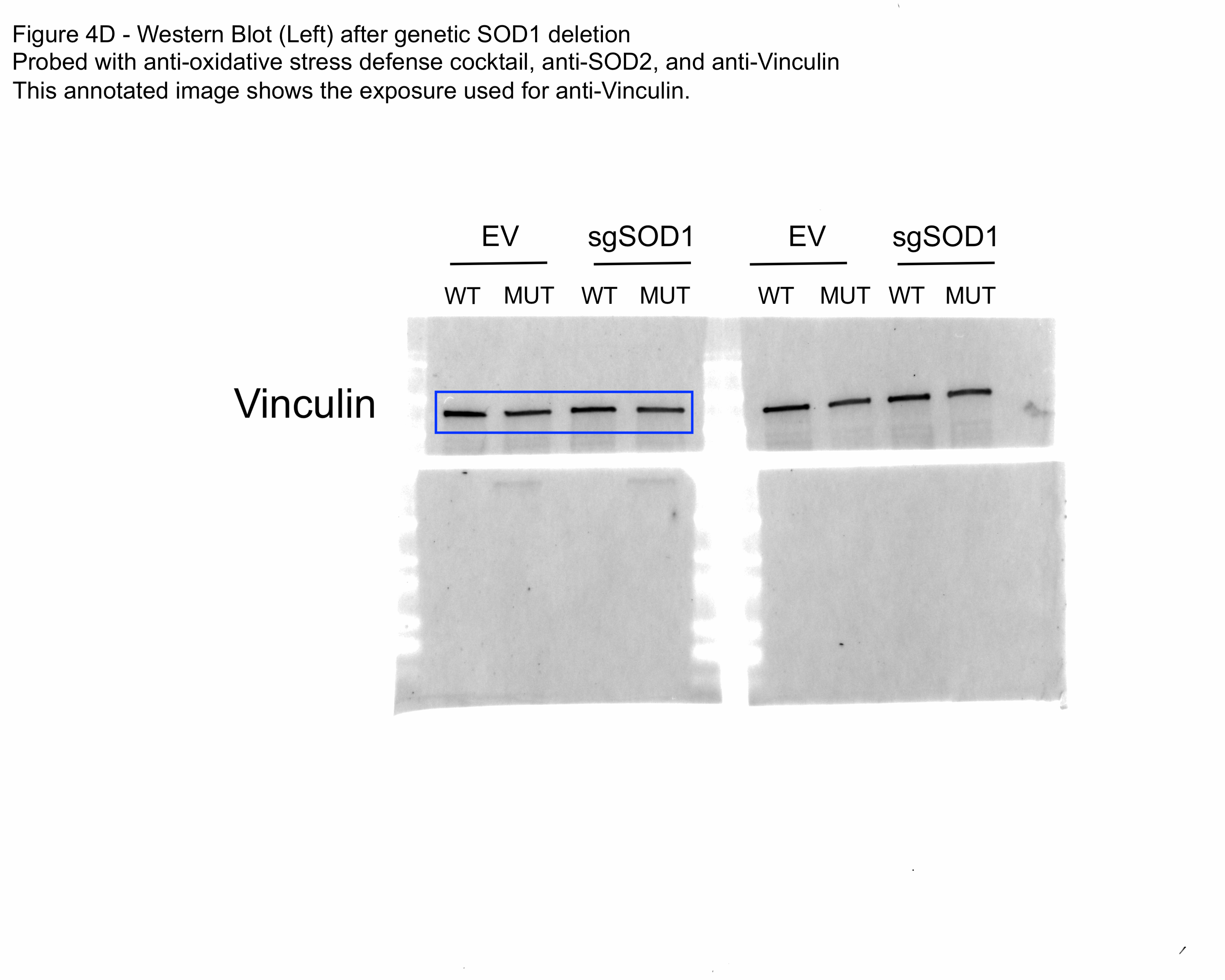

Supplement: Figure 4—source data 4. [file elife-91611-fig4-data4.zip › Figure 4-source data 4/SOD1-KO Western Blot (Left) - Annotated/Fig4D_ZhangL_Vinculin_An.tif]

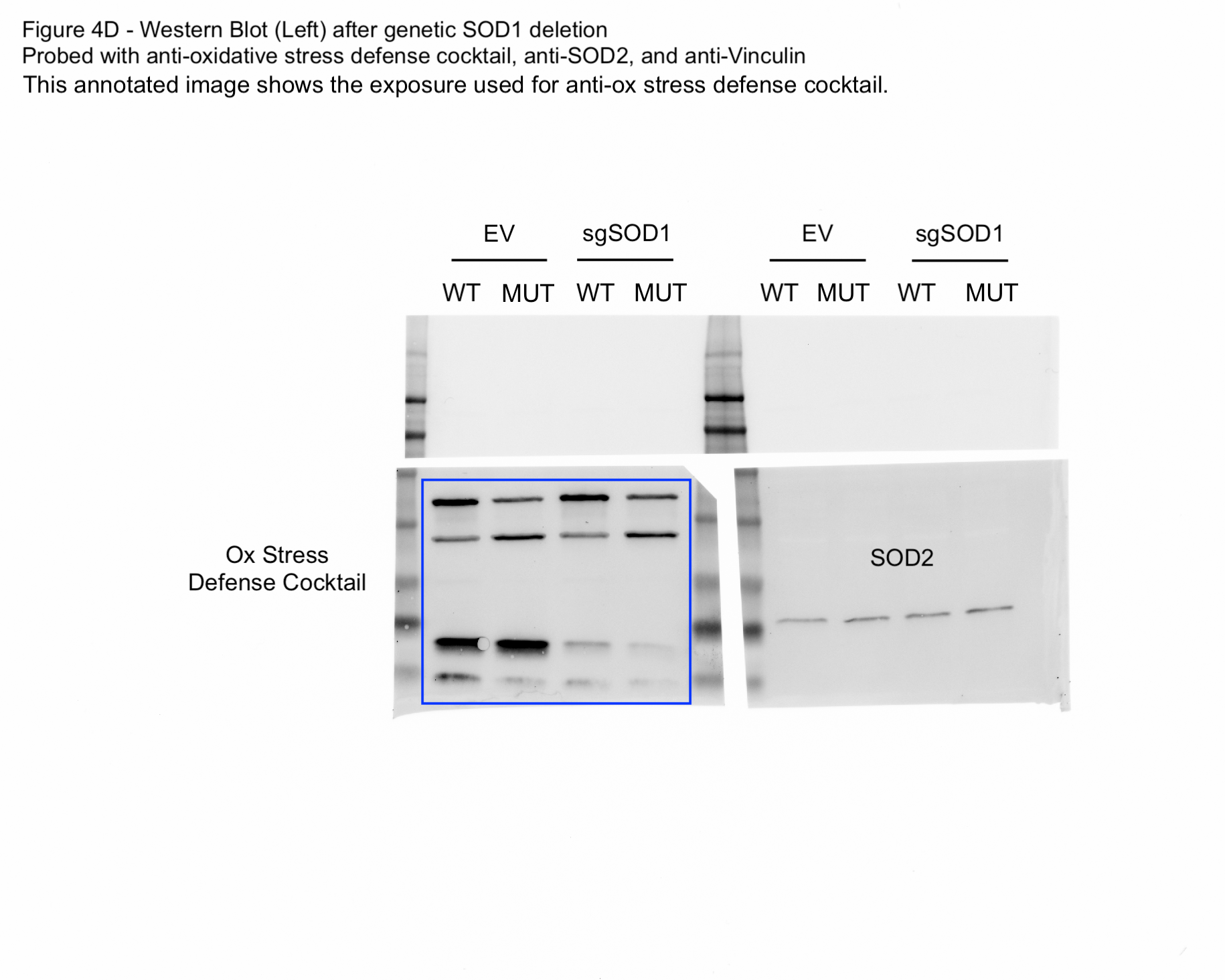

Supplement: Figure 4—source data 4. [file elife-91611-fig4-data4.zip › Figure 4-source data 4/SOD1-KO Western Blot (Left) - Annotated/Fig4D_ZhangL_OxStress_An.tif]

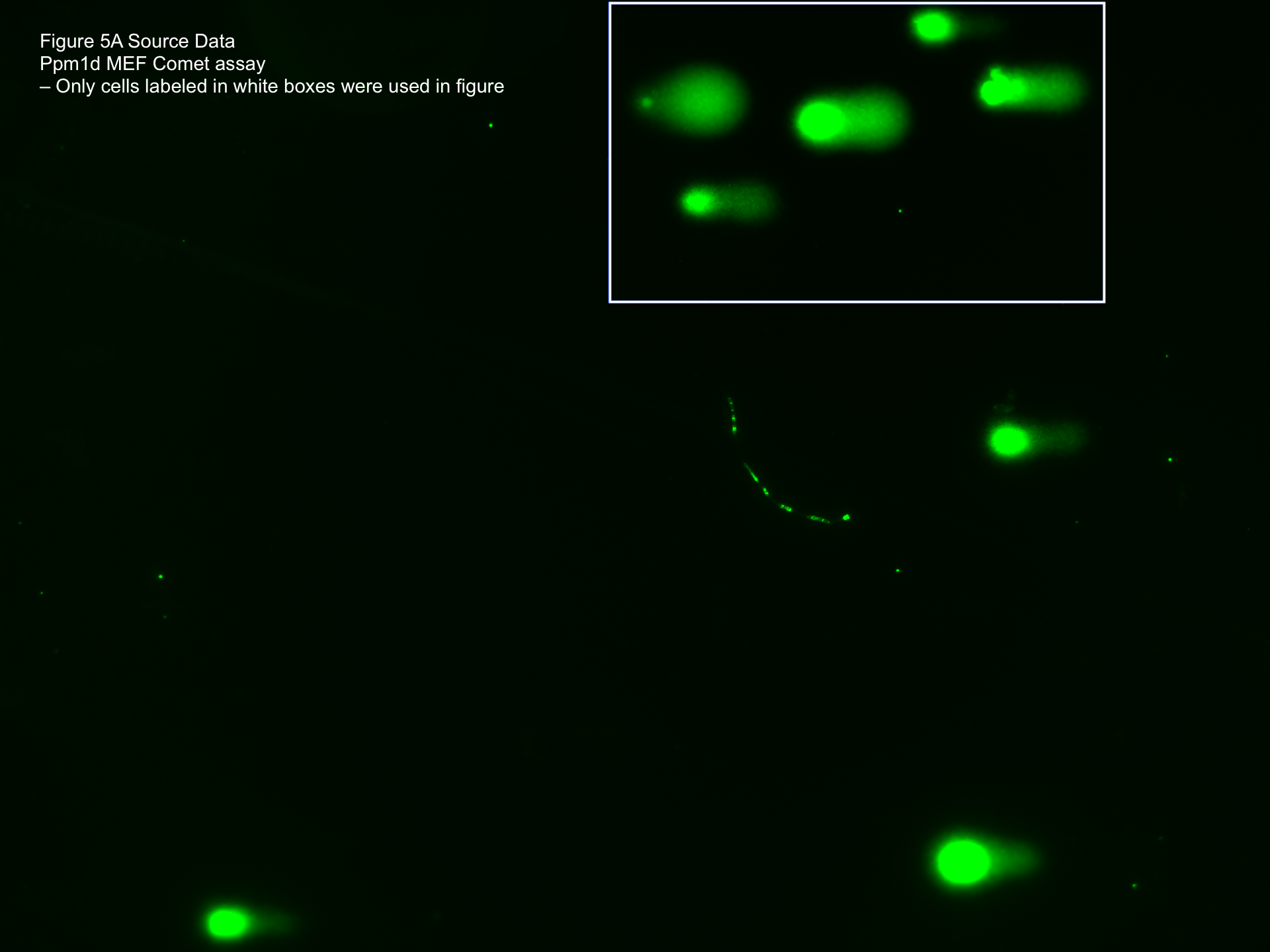

Supplement: Figure 5—source data 1. [file elife-91611-fig5-data1.zip › Figure 5-source data 1/Fig5A_ZhangL_Ppm1d_An.tif]

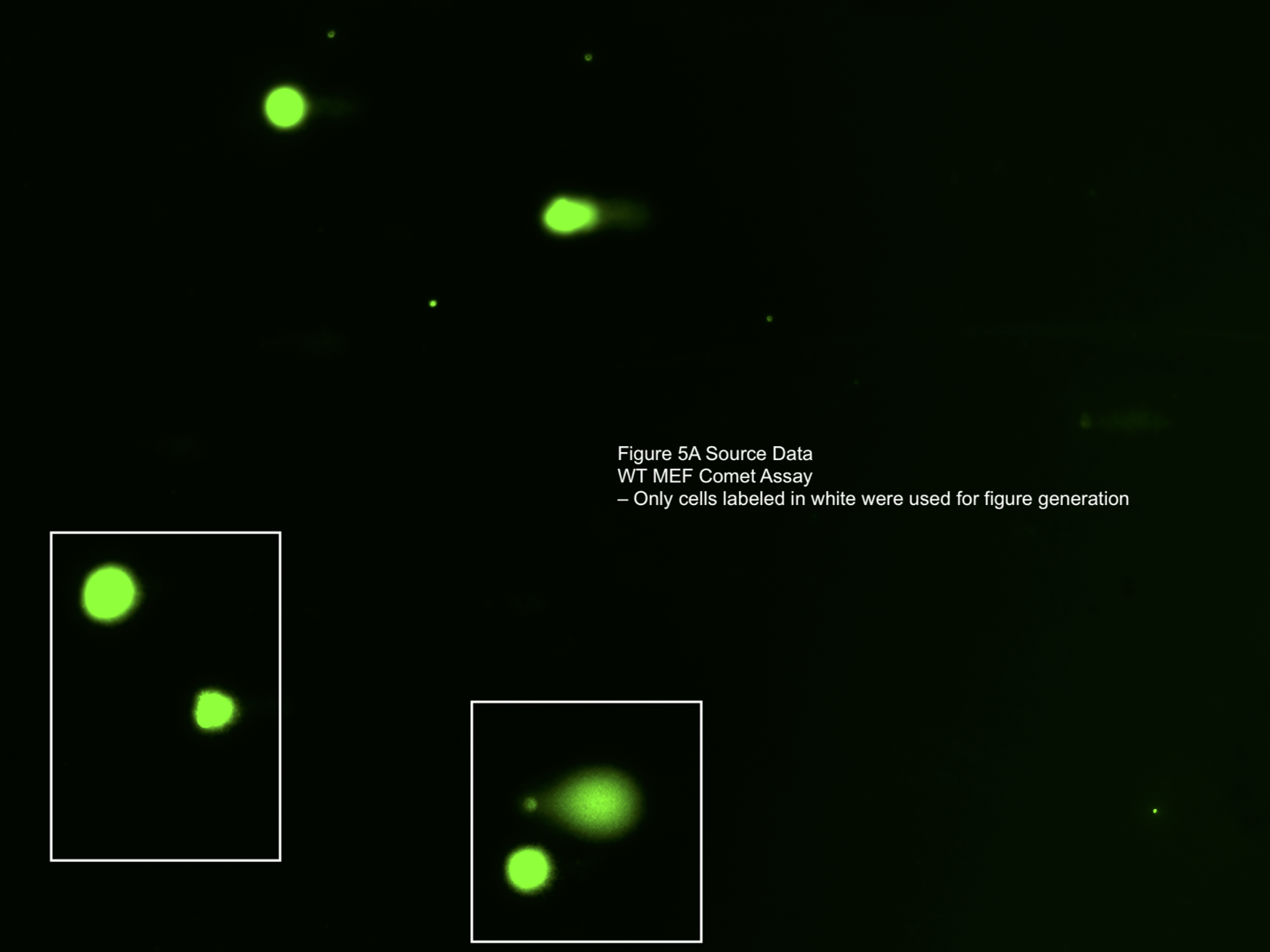

Supplement: Figure 5—source data 1. [file elife-91611-fig5-data1.zip › Figure 5-source data 1/Fig5A_ZhangL_WT_An.tif]

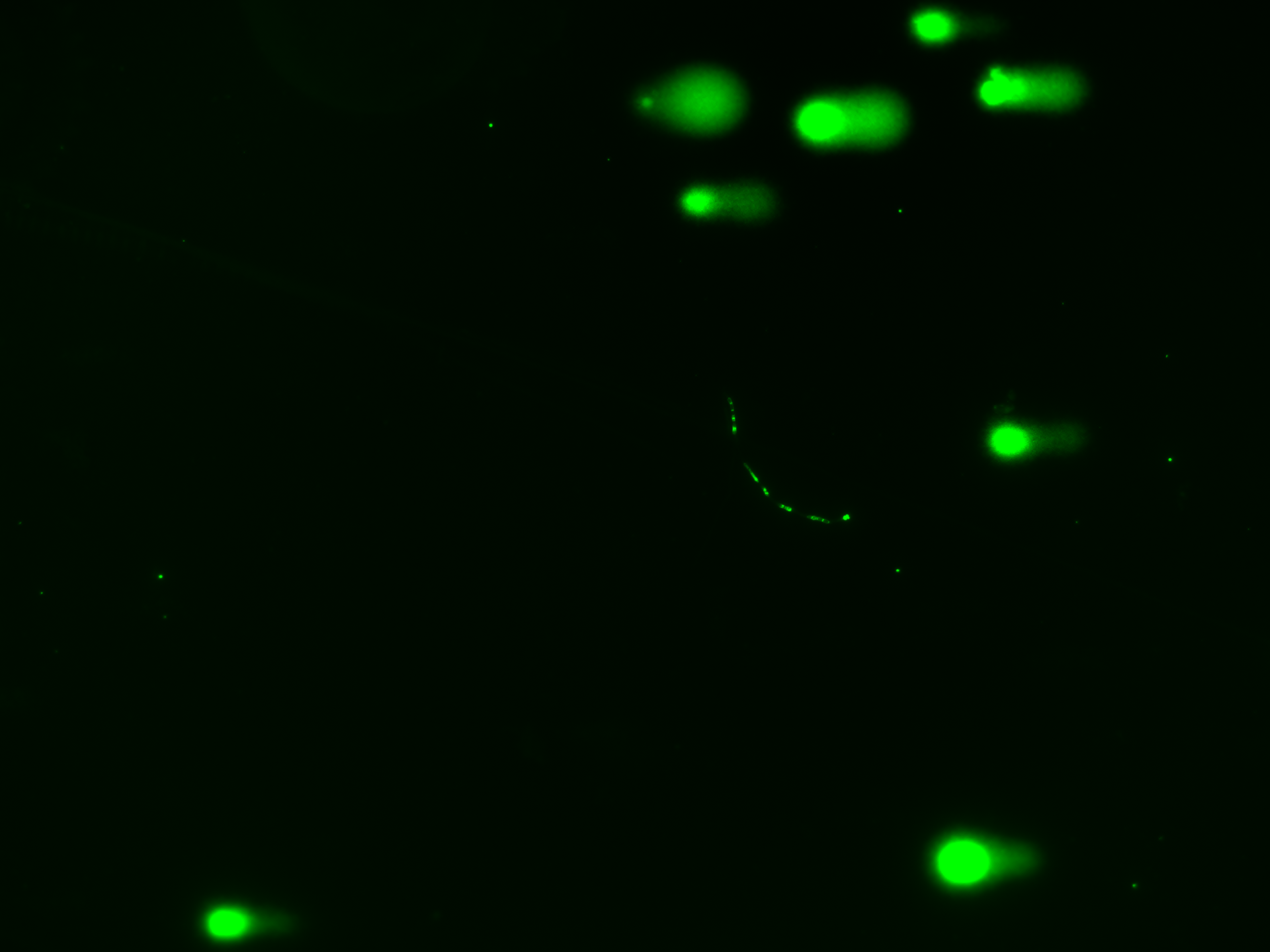

Supplement: Figure 5—source data 1. [file elife-91611-fig5-data1.zip › Figure 5-source data 1/Fig5A_ZhangL_Ppm1d_Raw.tif]

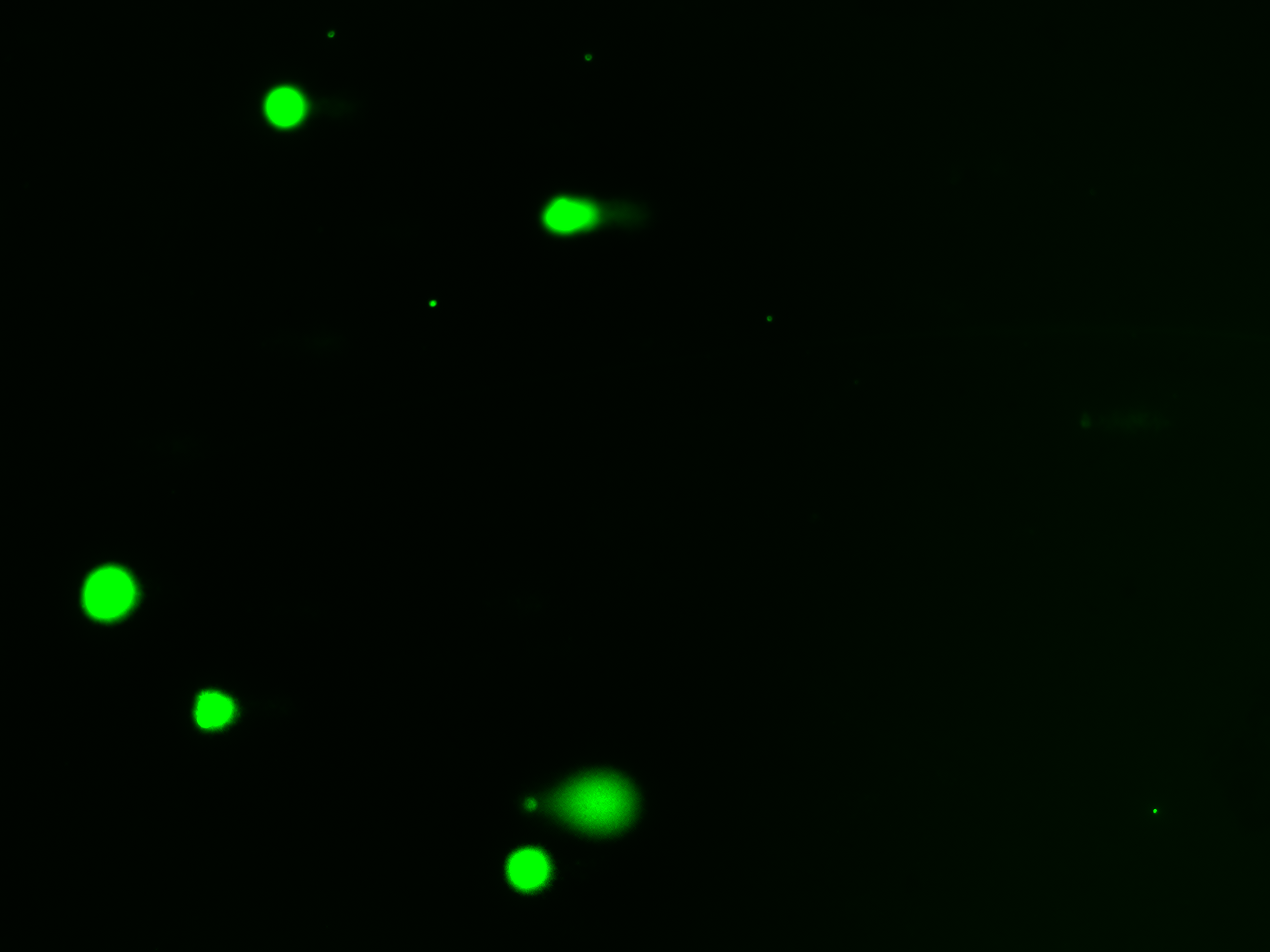

Supplement: Figure 5—source data 1. [file elife-91611-fig5-data1.zip › Figure 5-source data 1/Fig5A_ZhangL_WT_Raw.tif]

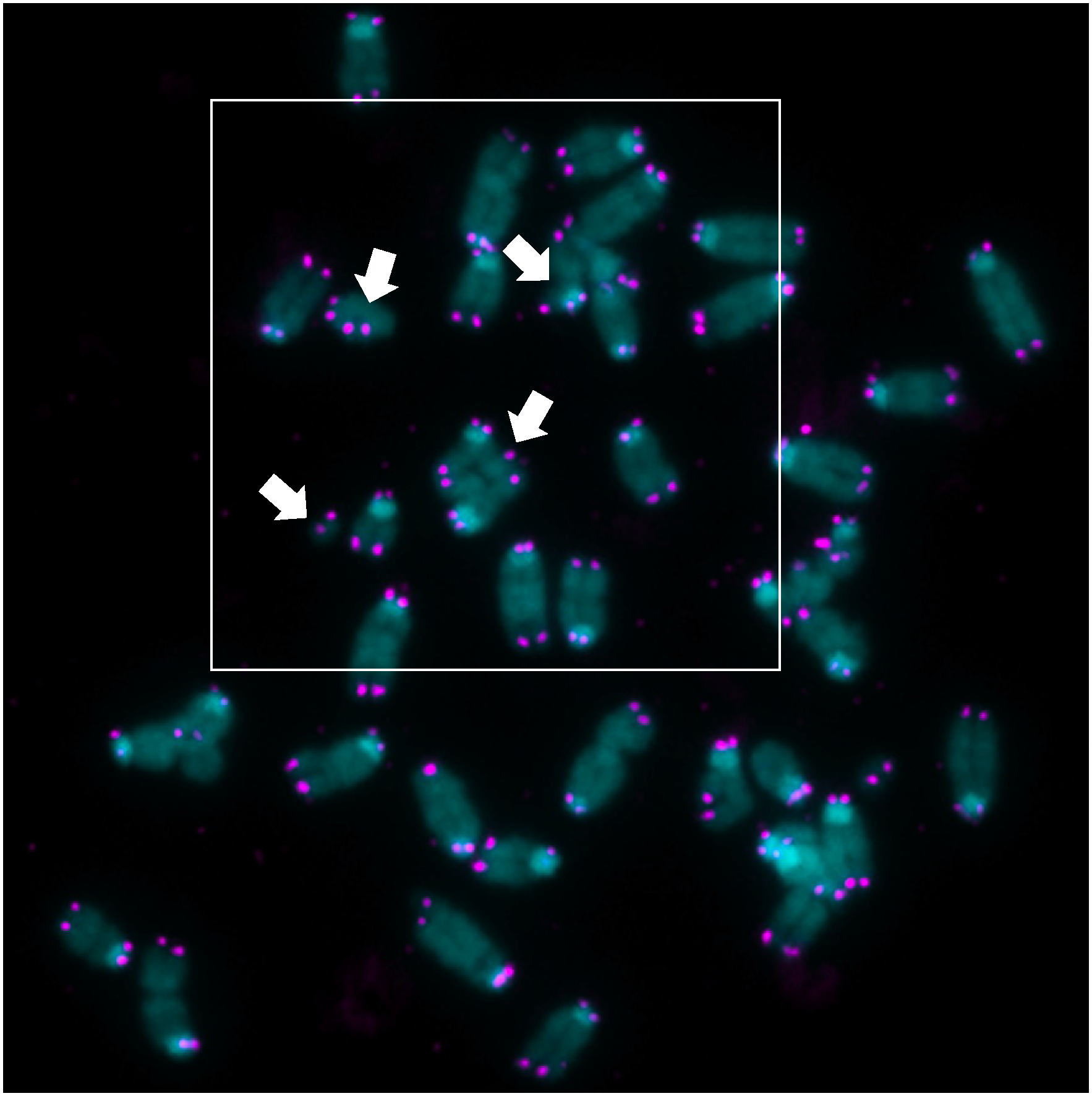

Supplement: Figure 5—source data 2. [file elife-91611-fig5-data2.zip › Figure 5-source data 2/Fig5C_ZhangL_An.tif]

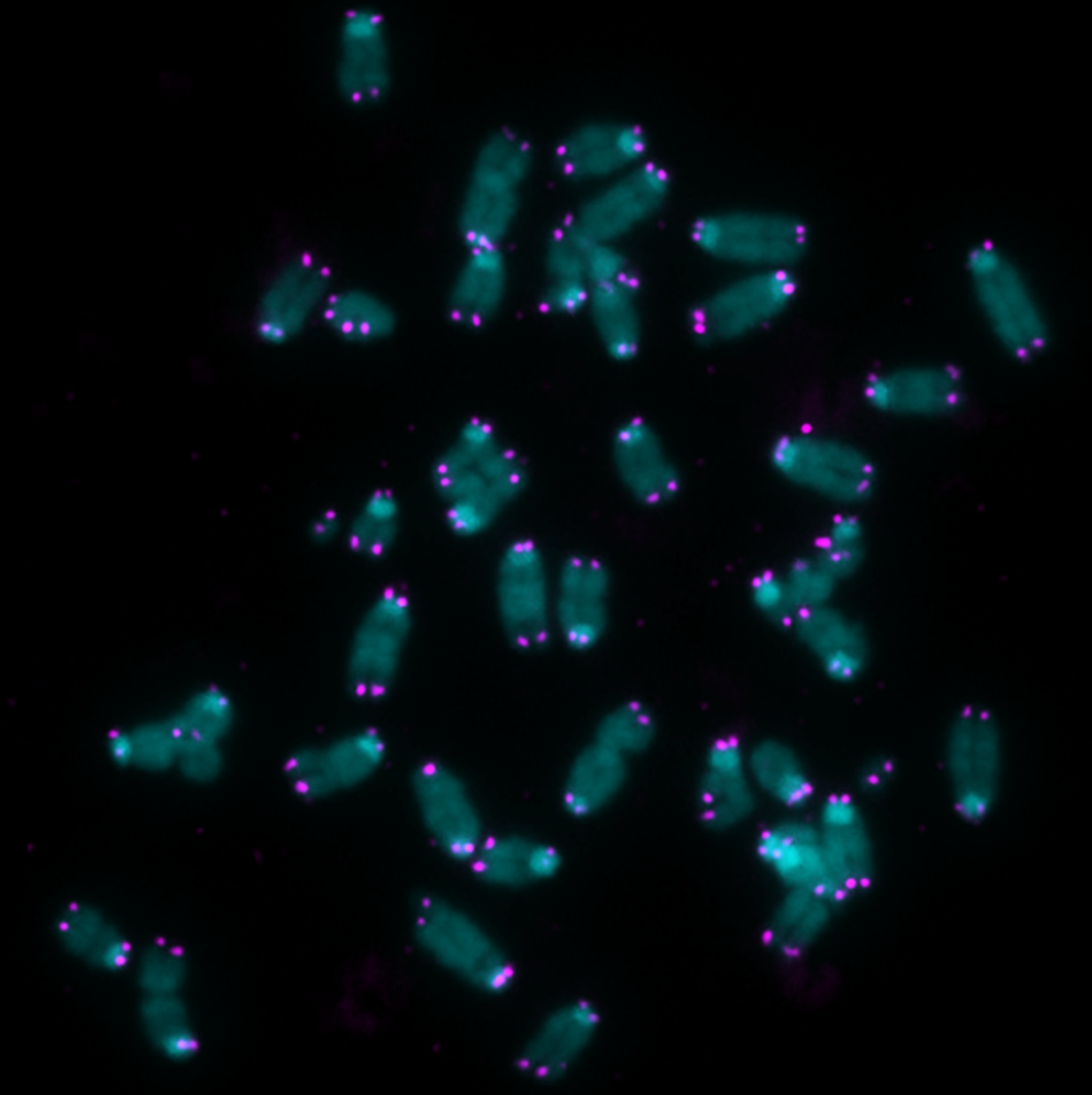

Supplement: Figure 5—source data 2. [file elife-91611-fig5-data2.zip › Figure 5-source data 2/Fig5C_ZhangL_Raw.png]

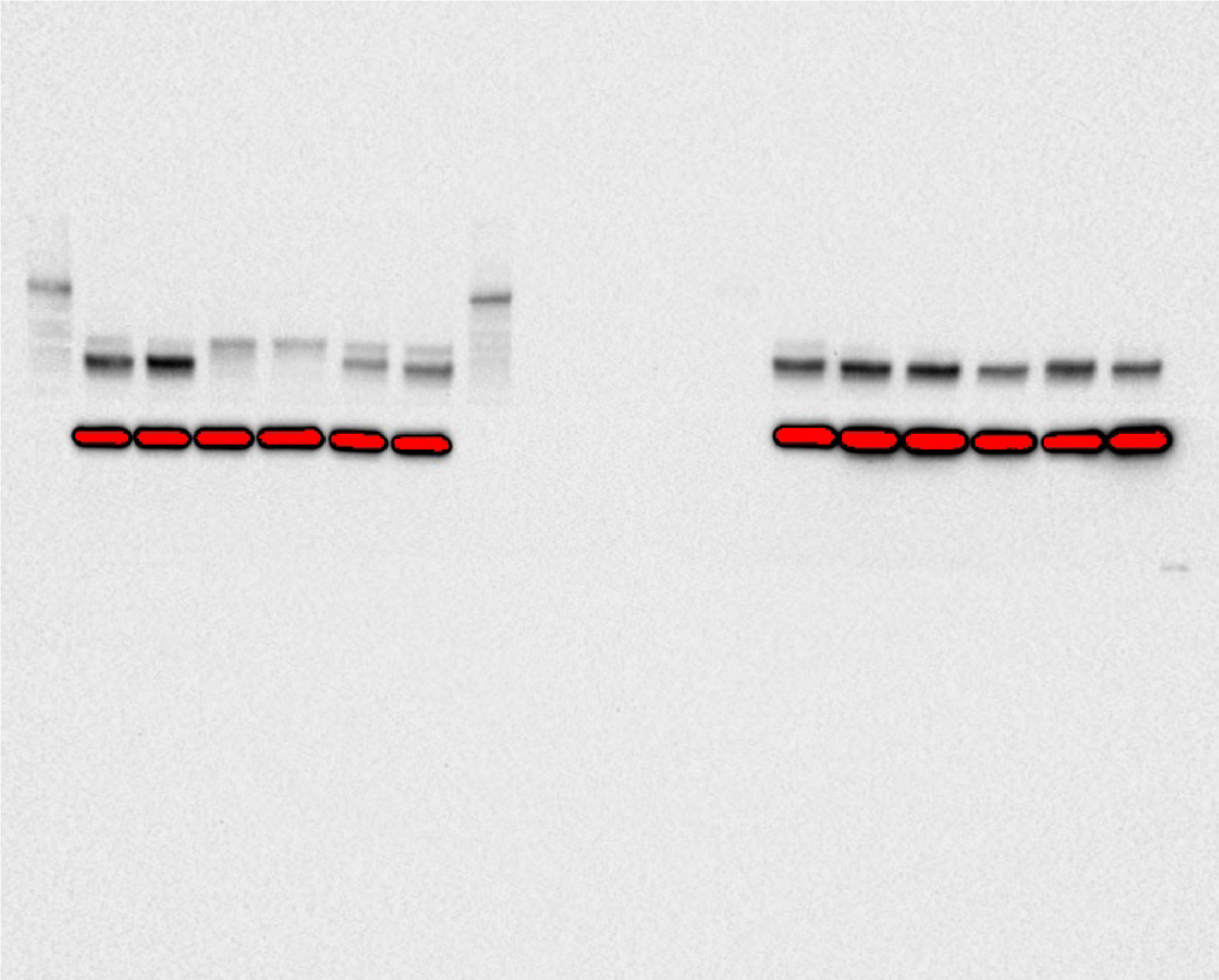

Supplement: Figure 5—figure supplement 1—source data 1. [file elife-91611-fig5-figsupp1-data1.zip › Figure 5-figure supplement 1-source data 1/Sup5A_ZhangL_PPM1D_Raw.tif]

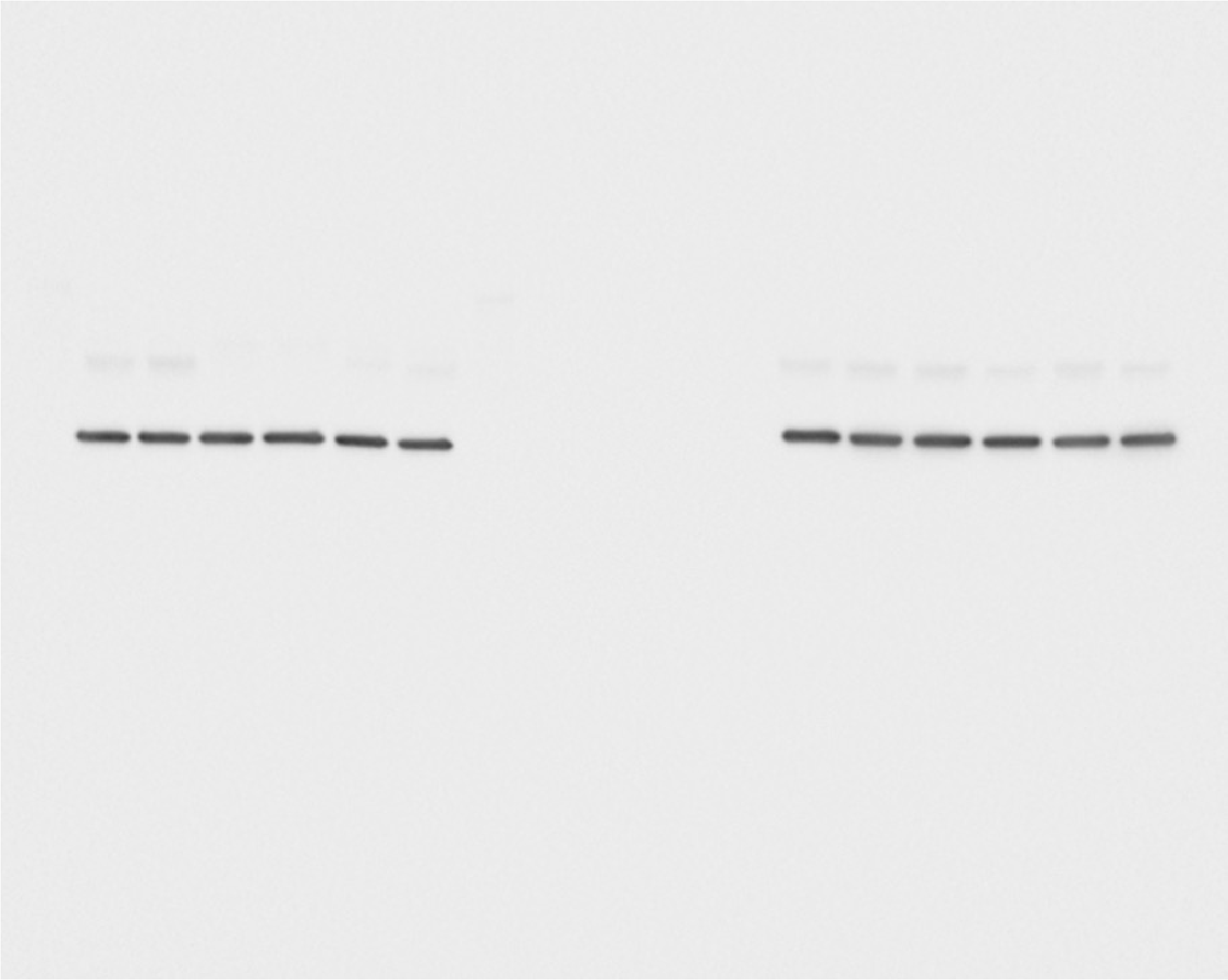

Supplement: Figure 5—figure supplement 1—source data 1. [file elife-91611-fig5-figsupp1-data1.zip › Figure 5-figure supplement 1-source data 1/Sup5A_ZhangL_GAPDH_Raw.tif]

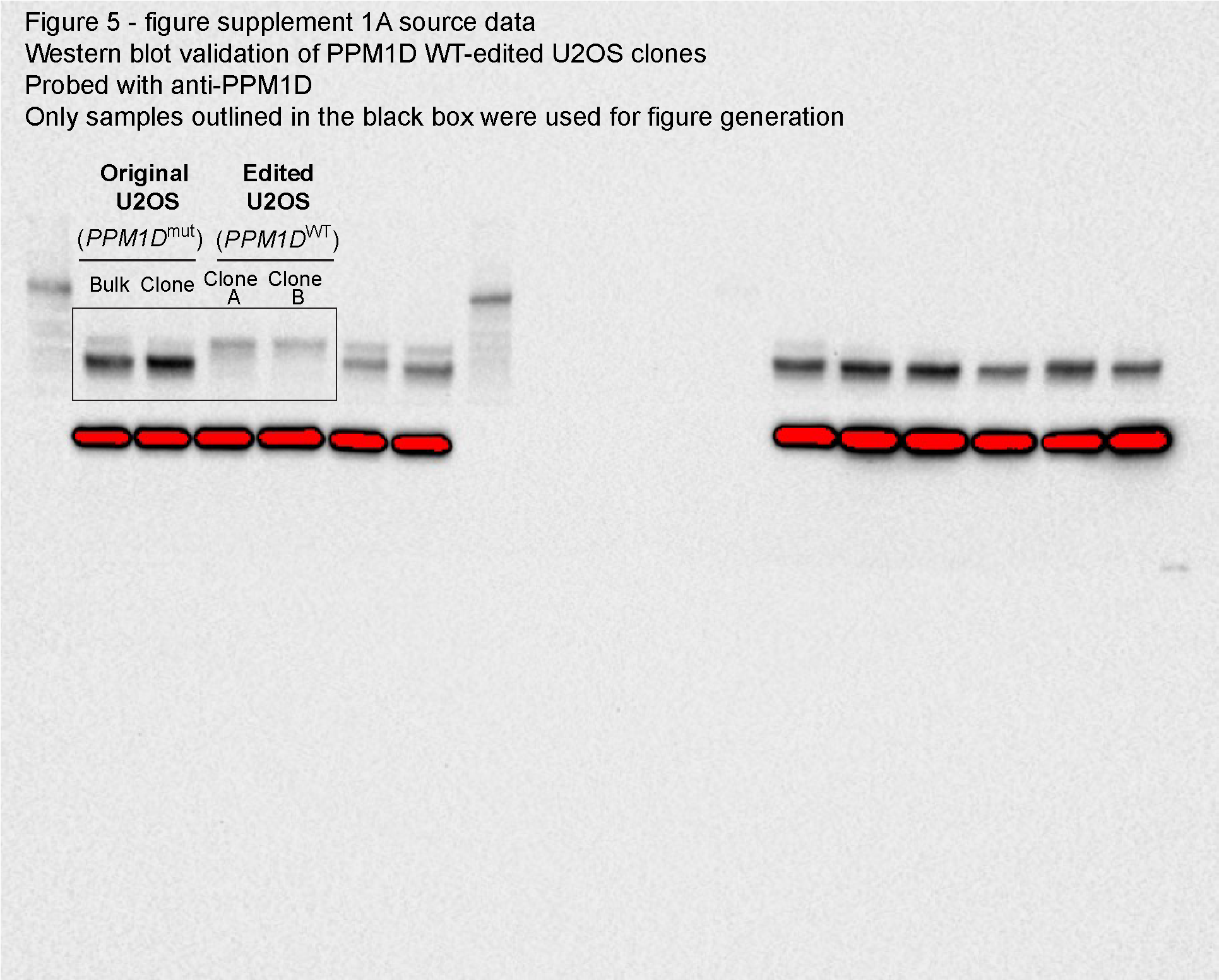

Supplement: Figure 5—figure supplement 1—source data 1. [file elife-91611-fig5-figsupp1-data1.zip › Figure 5-figure supplement 1-source data 1/Sup5A_ZhangL_PPM1D_An.tif]

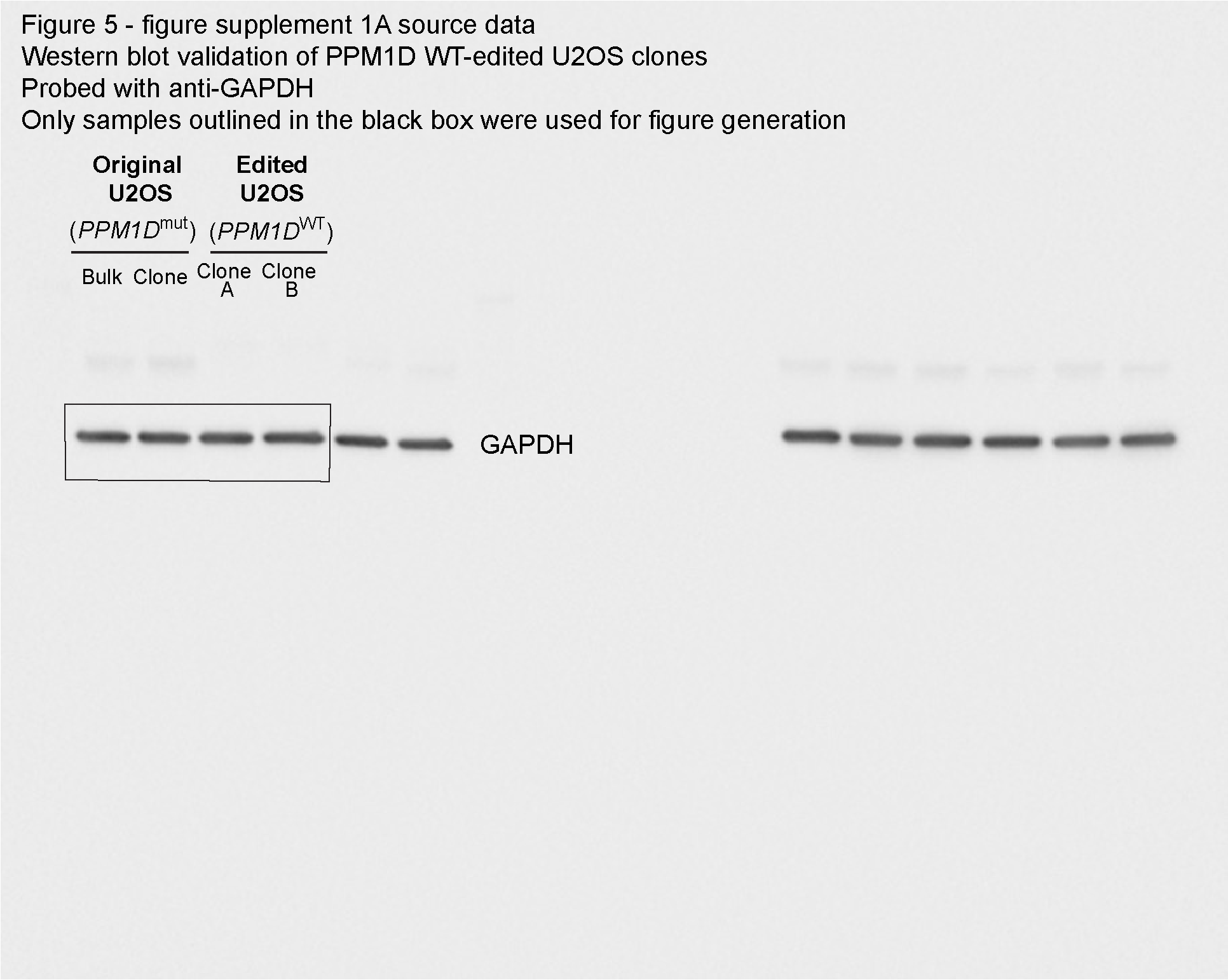

Supplement: Figure 5—figure supplement 1—source data 1. [file elife-91611-fig5-figsupp1-data1.zip › Figure 5-figure supplement 1-source data 1/Sup5A_ZhangL_GAPDH_An.tif]

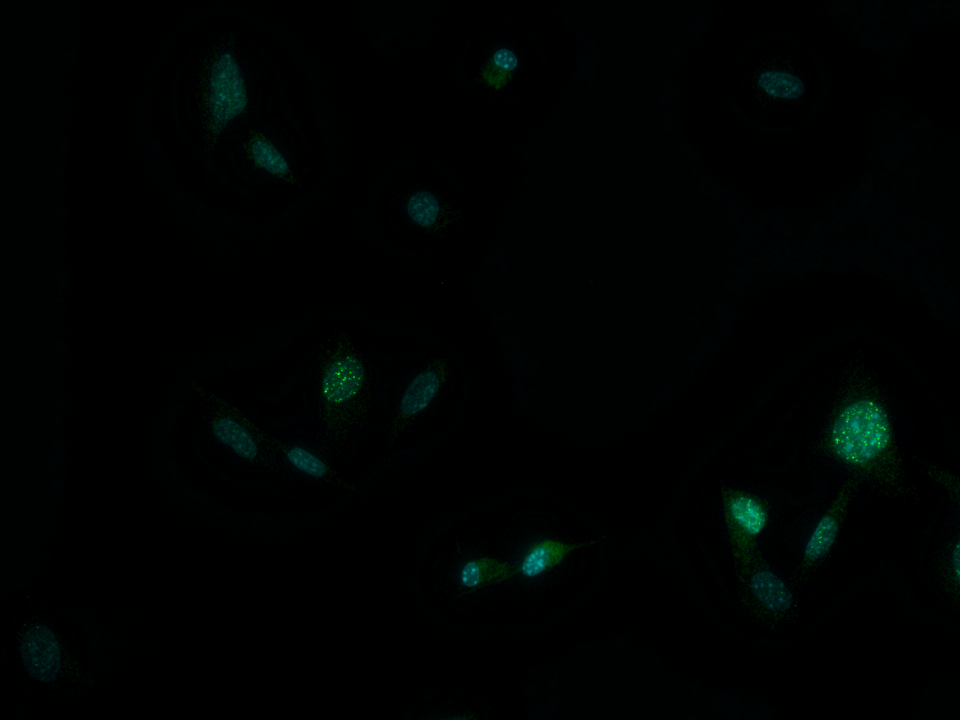

Supplement: Figure 5—figure supplement 1—source data 2. [file elife-91611-fig5-figsupp1-data2.zip › Figure 5-figure supplement 1-source data 2/Sup5B_ZhangL_Ppm1d_Overlay_Raw.tif]

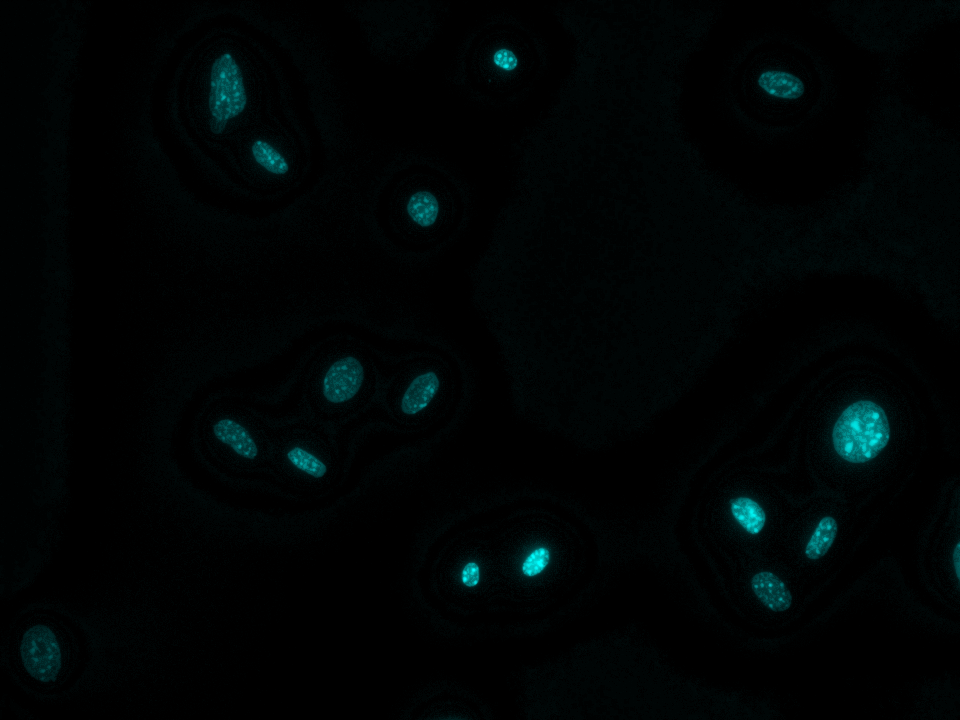

Supplement: Figure 5—figure supplement 1—source data 2. [file elife-91611-fig5-figsupp1-data2.zip › Figure 5-figure supplement 1-source data 2/Sup5B_ZhangL_Ppm1d_DAPI_Raw.tif]

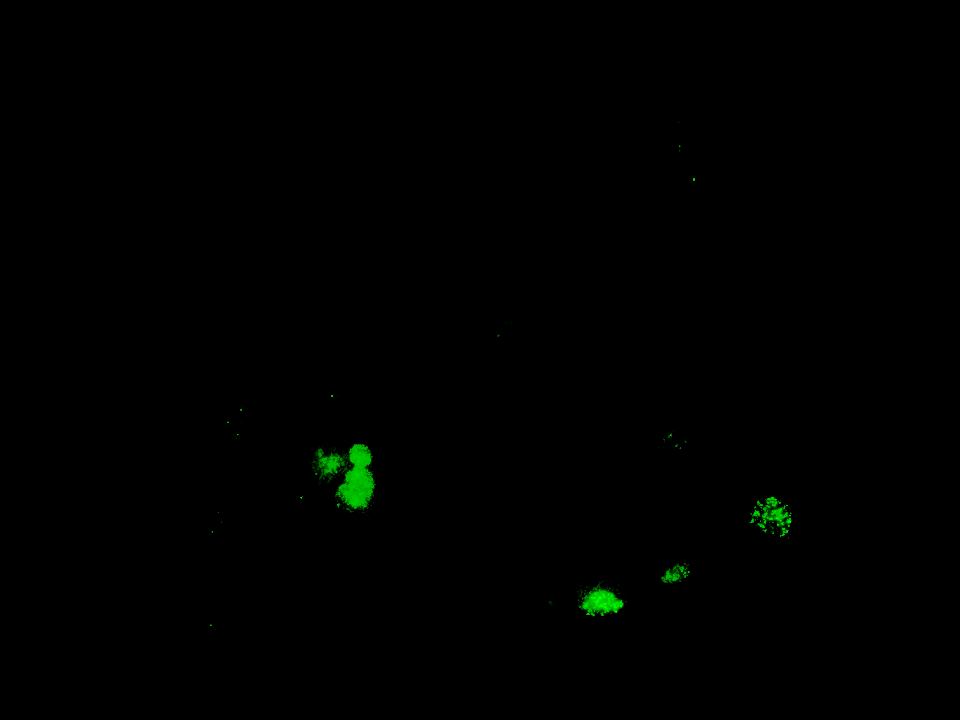

Supplement: Figure 5—figure supplement 1—source data 2. [file elife-91611-fig5-figsupp1-data2.zip › Figure 5-figure supplement 1-source data 2/Sup5B_ZhangL_WT_Rad51_Raw.tif]

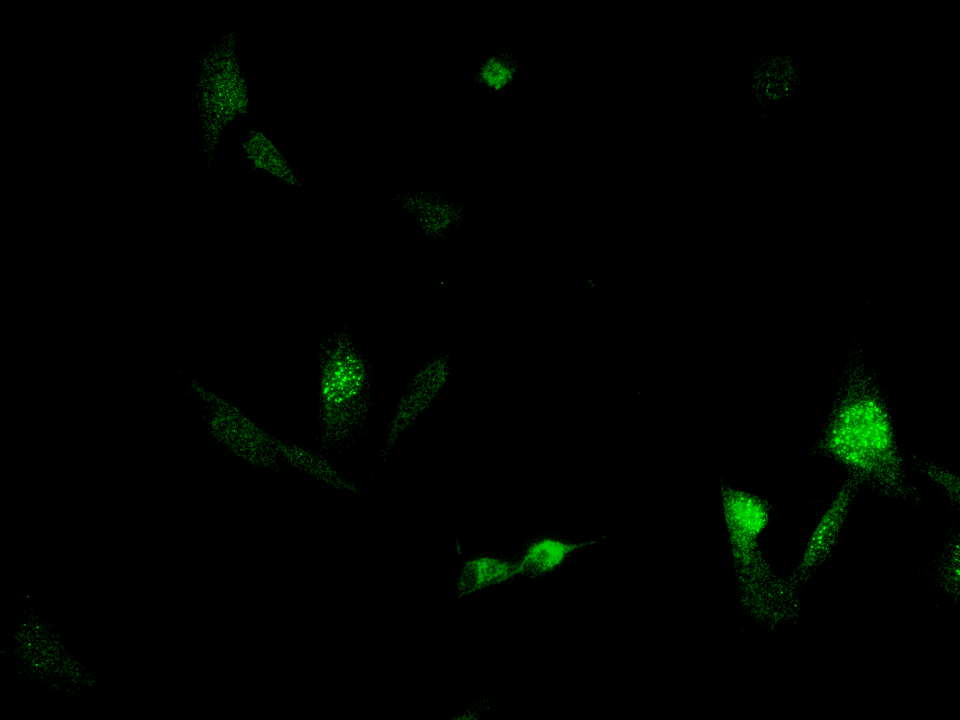

Supplement: Figure 5—figure supplement 1—source data 2. [file elife-91611-fig5-figsupp1-data2.zip › Figure 5-figure supplement 1-source data 2/Sup5B_ZhangL_Ppm1d_Rad51_Raw.tif]

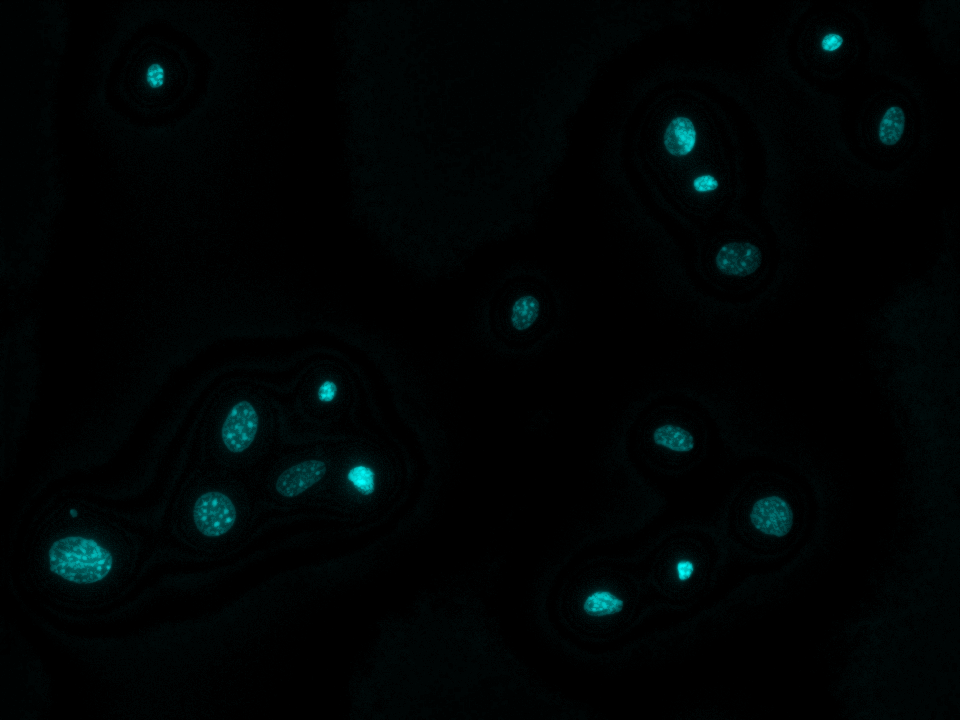

Supplement: Figure 5—figure supplement 1—source data 2. [file elife-91611-fig5-figsupp1-data2.zip › Figure 5-figure supplement 1-source data 2/Sup5B_ZhangL_WT_DAPI_Raw.tif]

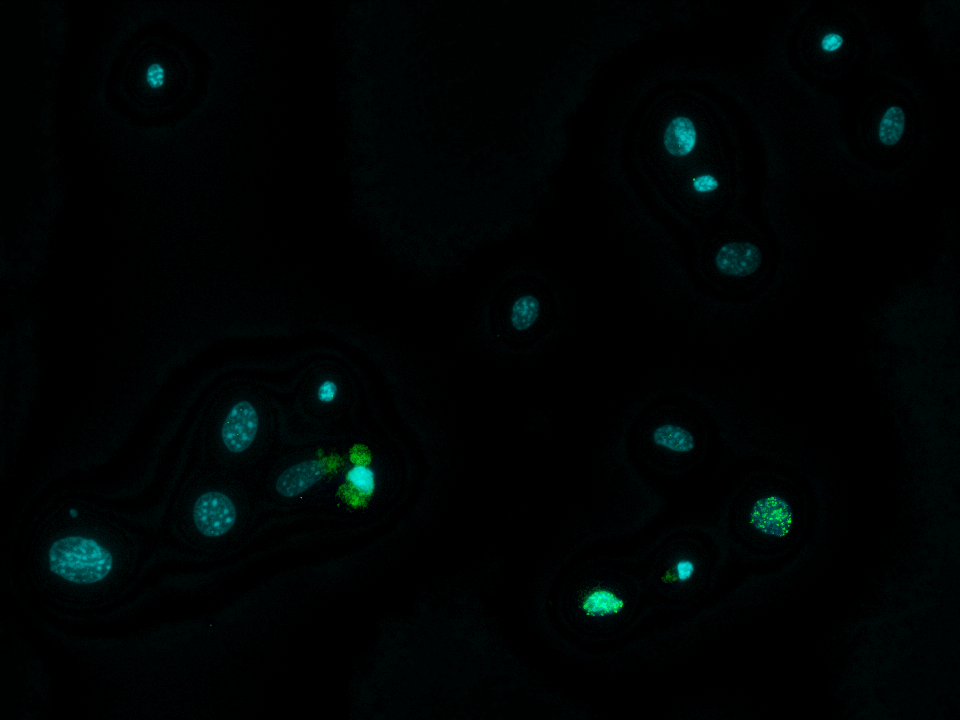

Supplement: Figure 5—figure supplement 1—source data 2. [file elife-91611-fig5-figsupp1-data2.zip › Figure 5-figure supplement 1-source data 2/Sup5B_ZhangL_WT_Overlay_Raw.tif]

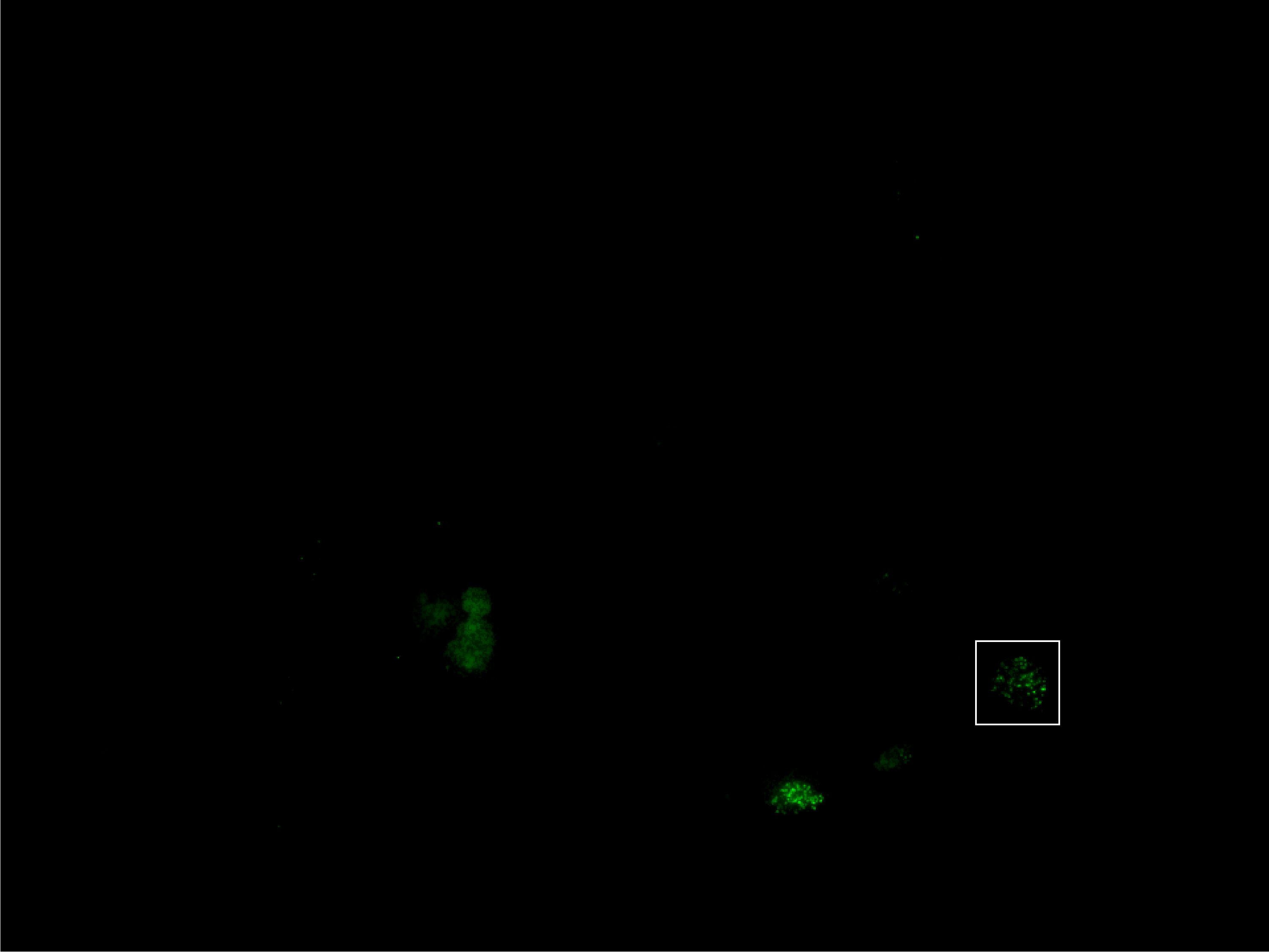

Supplement: Figure 5—figure supplement 1—source data 2. [file elife-91611-fig5-figsupp1-data2.zip › Figure 5-figure supplement 1-source data 2/Supp 5B Annotated Files/Sup5B_ZhangL_WT_Rad51_An.tif]

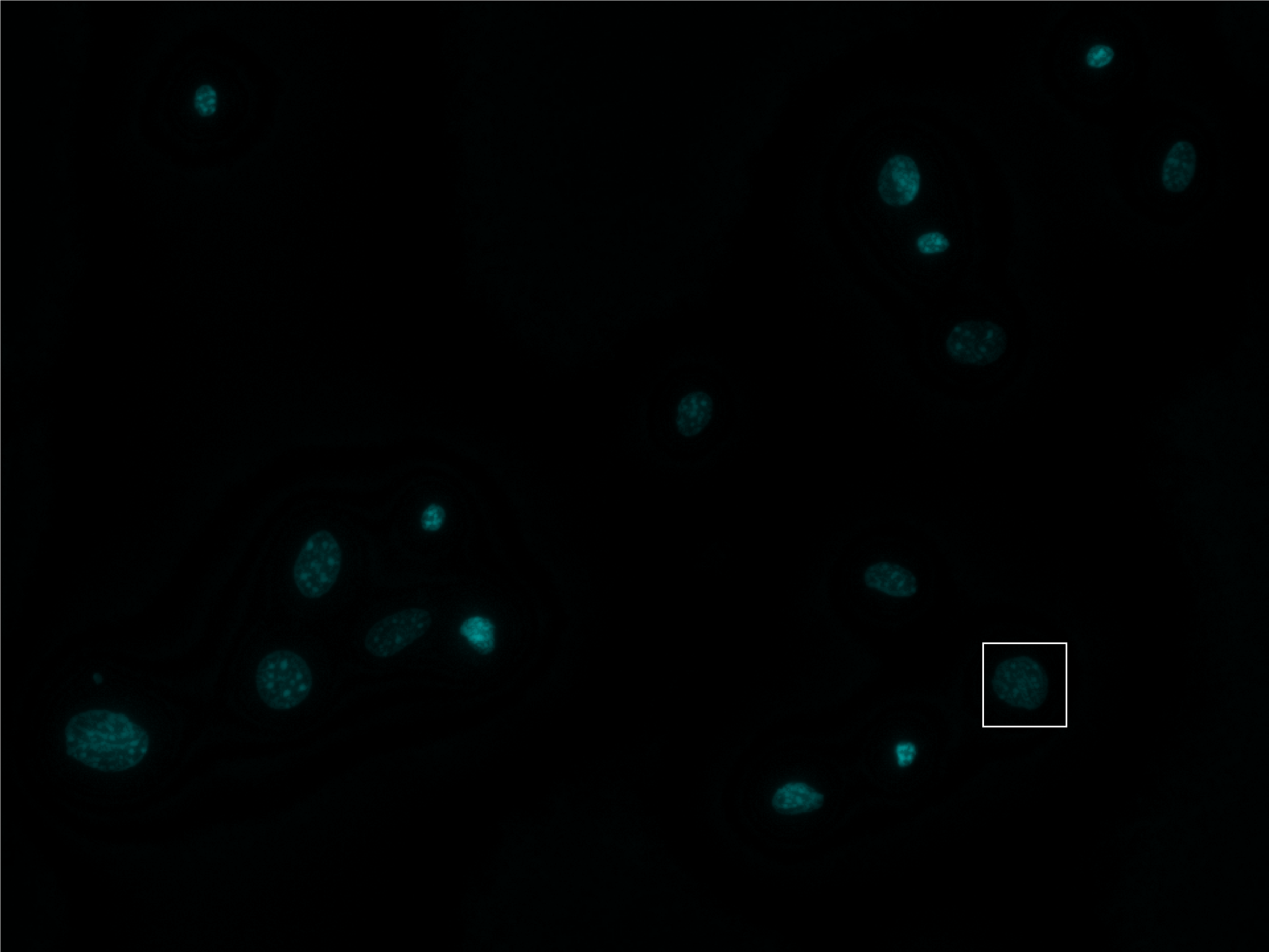

Supplement: Figure 5—figure supplement 1—source data 2. [file elife-91611-fig5-figsupp1-data2.zip › Figure 5-figure supplement 1-source data 2/Supp 5B Annotated Files/Sup5B_ZhangL_WT_DAPI_An.tif]

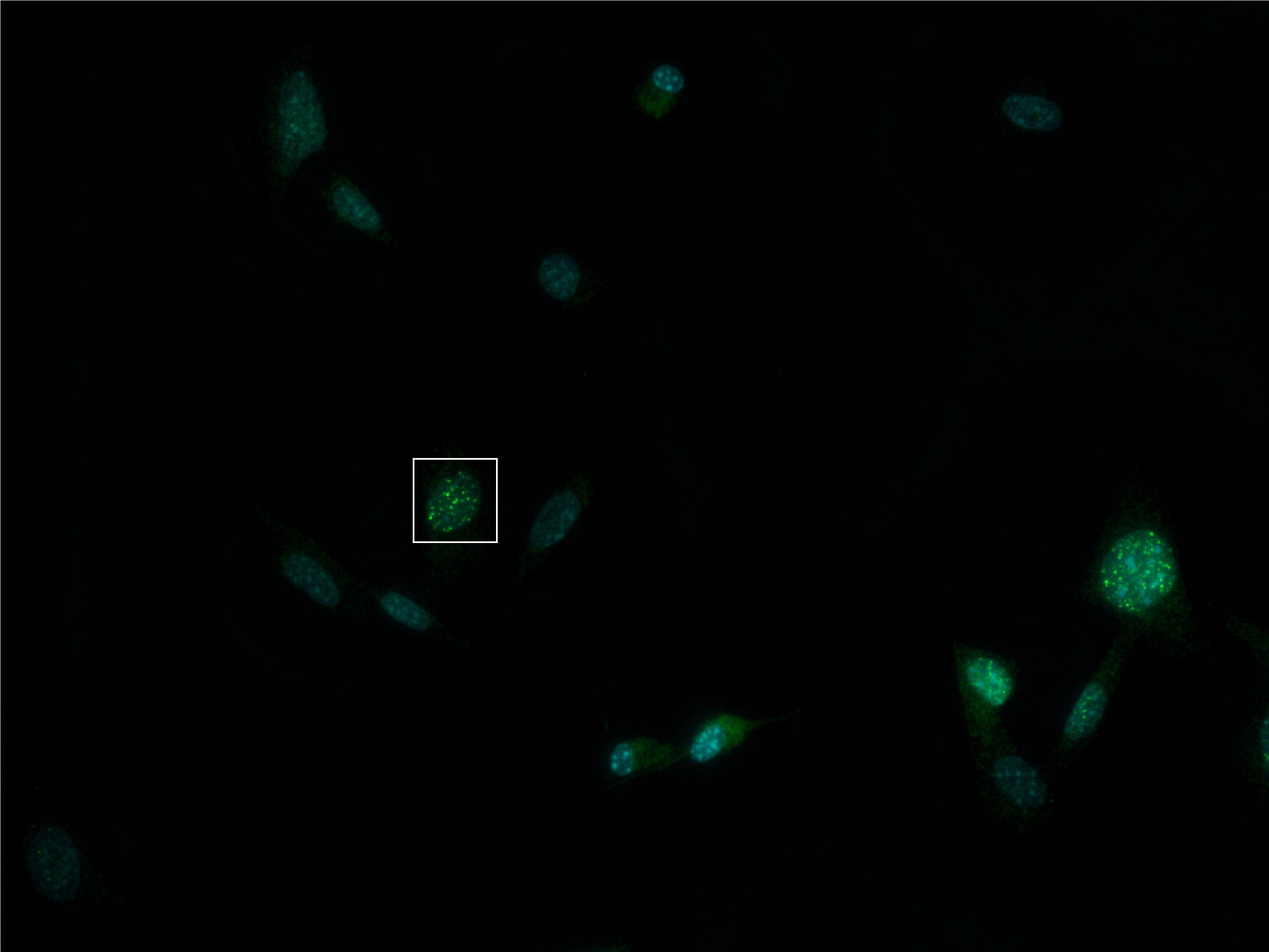

Supplement: Figure 5—figure supplement 1—source data 2. [file elife-91611-fig5-figsupp1-data2.zip › Figure 5-figure supplement 1-source data 2/Supp 5B Annotated Files/Sup5B_ZhangL_Ppm1d_Overlay_An.tif]

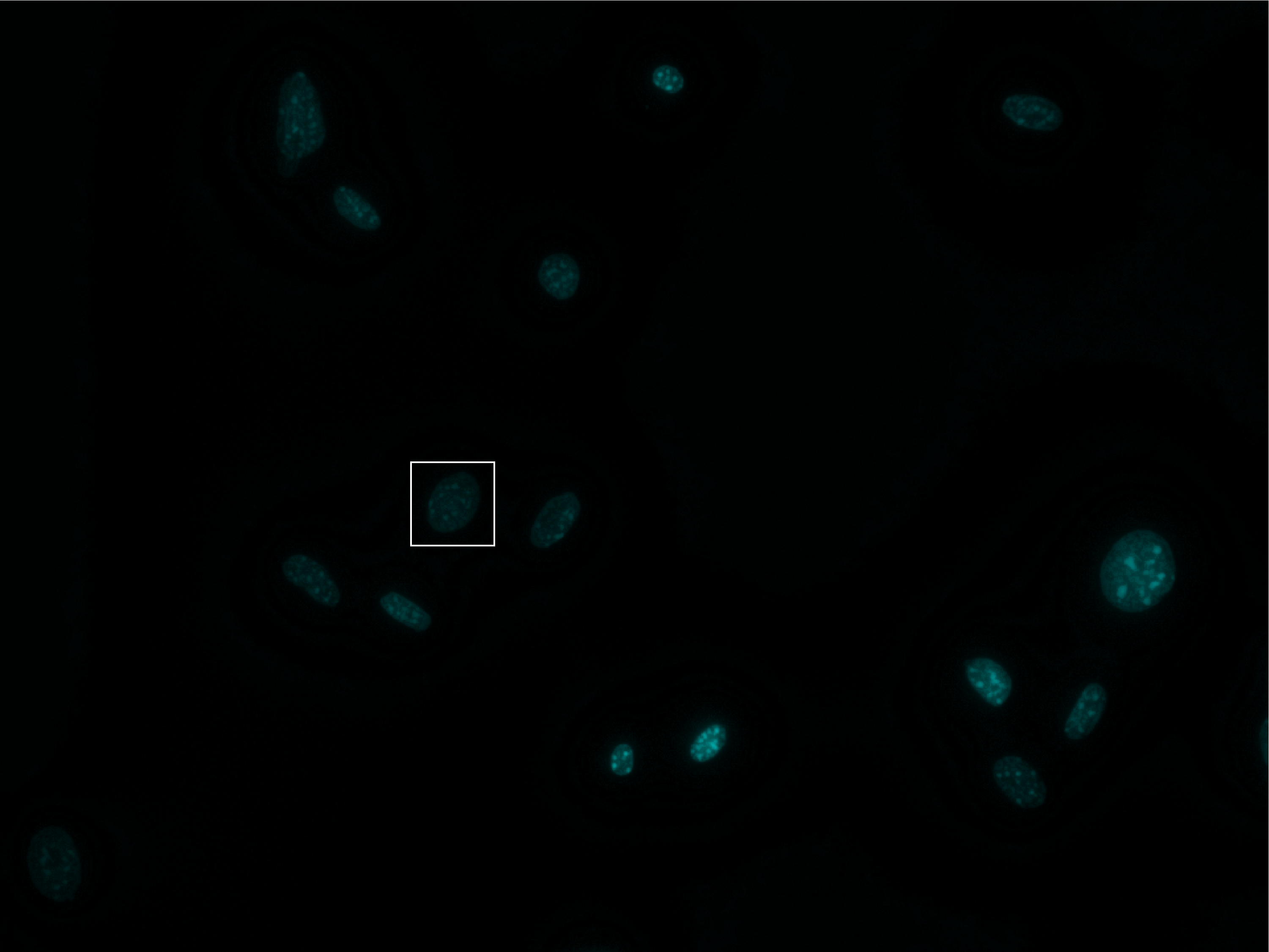

Supplement: Figure 5—figure supplement 1—source data 2. [file elife-91611-fig5-figsupp1-data2.zip › Figure 5-figure supplement 1-source data 2/Supp 5B Annotated Files/Sup5B_ZhangL_Ppm1d_DAPI_An.tif]

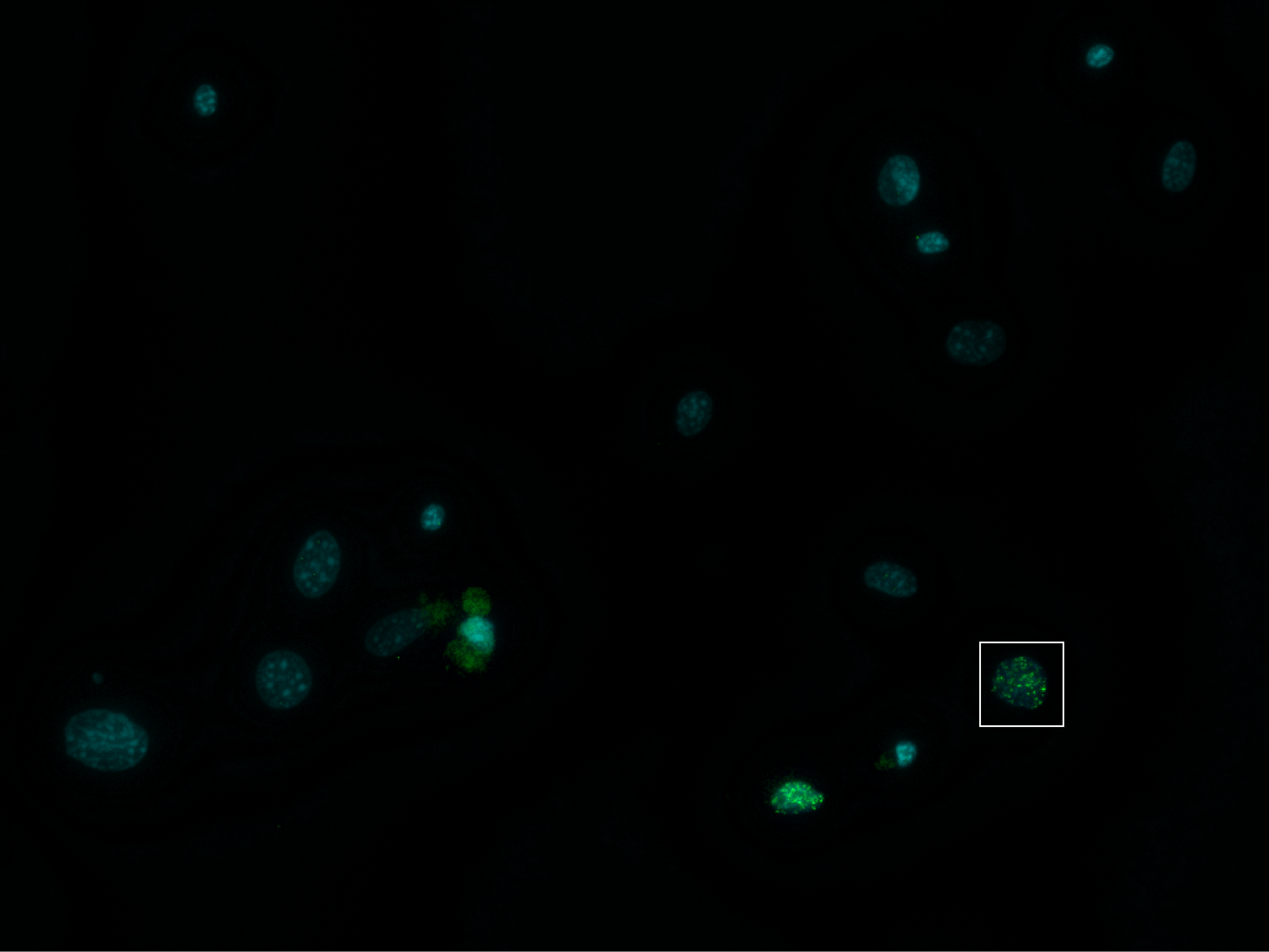

Supplement: Figure 5—figure supplement 1—source data 2. [file elife-91611-fig5-figsupp1-data2.zip › Figure 5-figure supplement 1-source data 2/Supp 5B Annotated Files/Sup5B_ZhangL_WT_Overlay_An.tif]

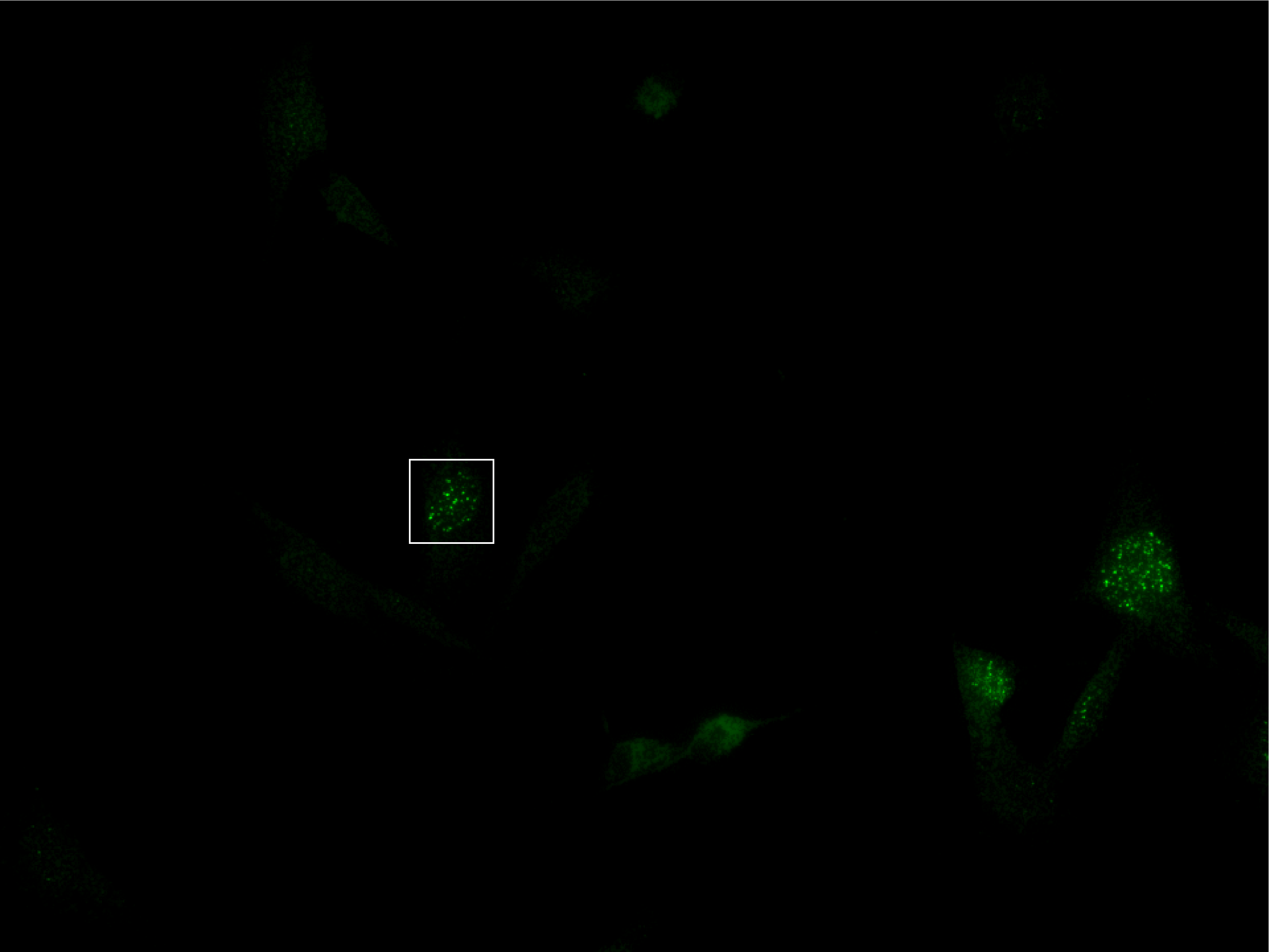

Supplement: Figure 5—figure supplement 1—source data 2. [file elife-91611-fig5-figsupp1-data2.zip › Figure 5-figure supplement 1-source data 2/Supp 5B Annotated Files/Sup5B_ZhangL_Ppm1d_Rad51_An.tif]

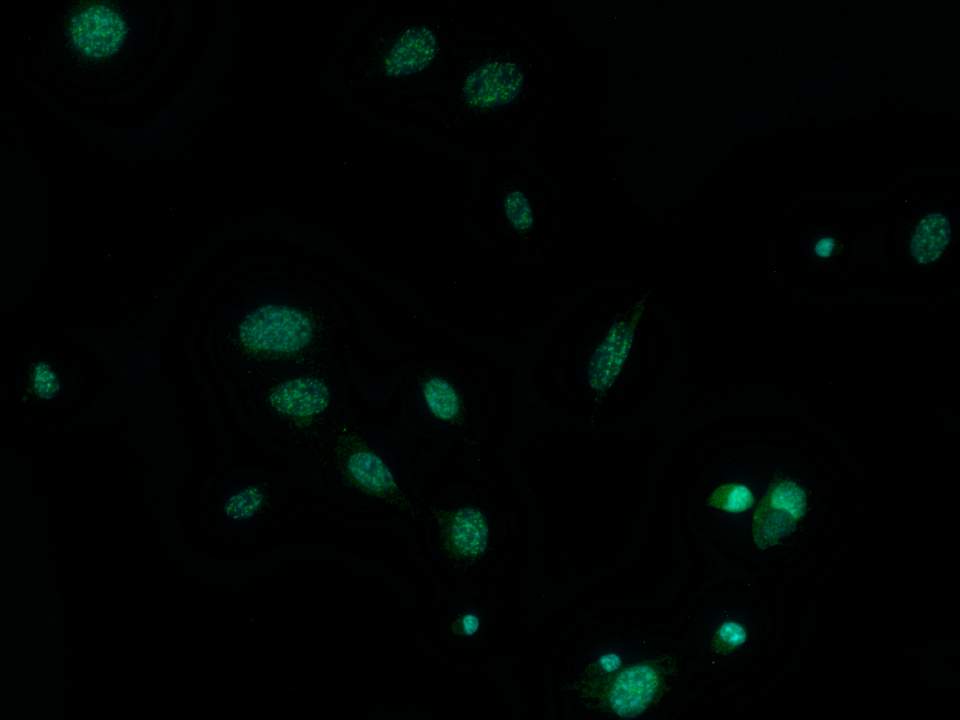

Supplement: Figure 5—figure supplement 1—source data 3. [file elife-91611-fig5-figsupp1-data3.zip › Figure 5-figure supplement 1-source data 3/Sup5C_ZhangL_WT_Overlay_Raw.tif]

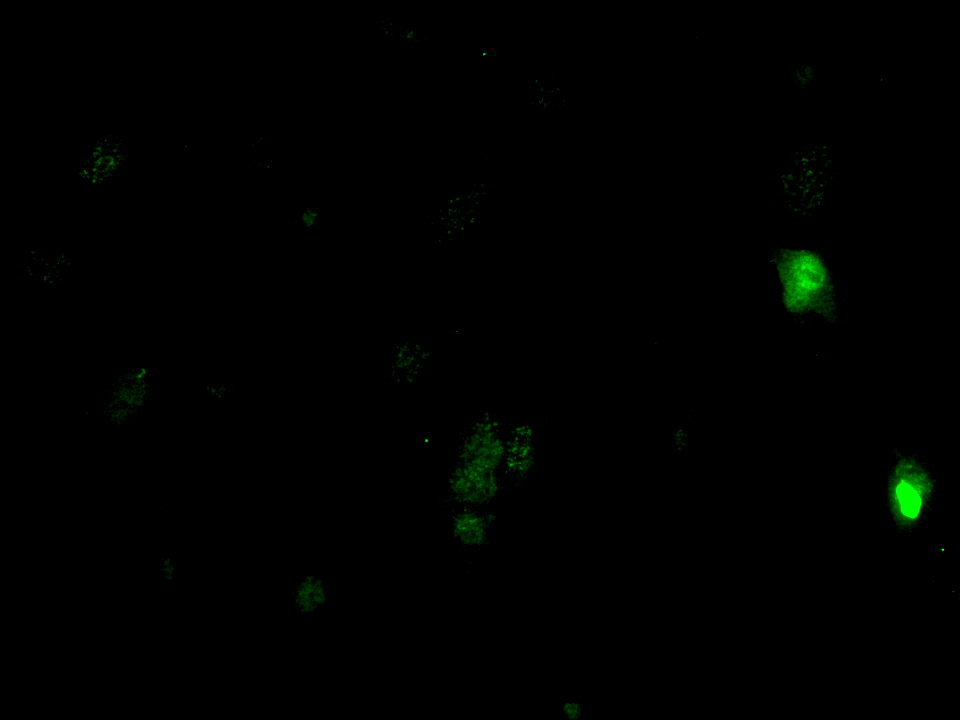

Supplement: Figure 5—figure supplement 1—source data 3. [file elife-91611-fig5-figsupp1-data3.zip › Figure 5-figure supplement 1-source data 3/Sup5C_ZhangL_Ppm1d_53BP1_Raw.tif]

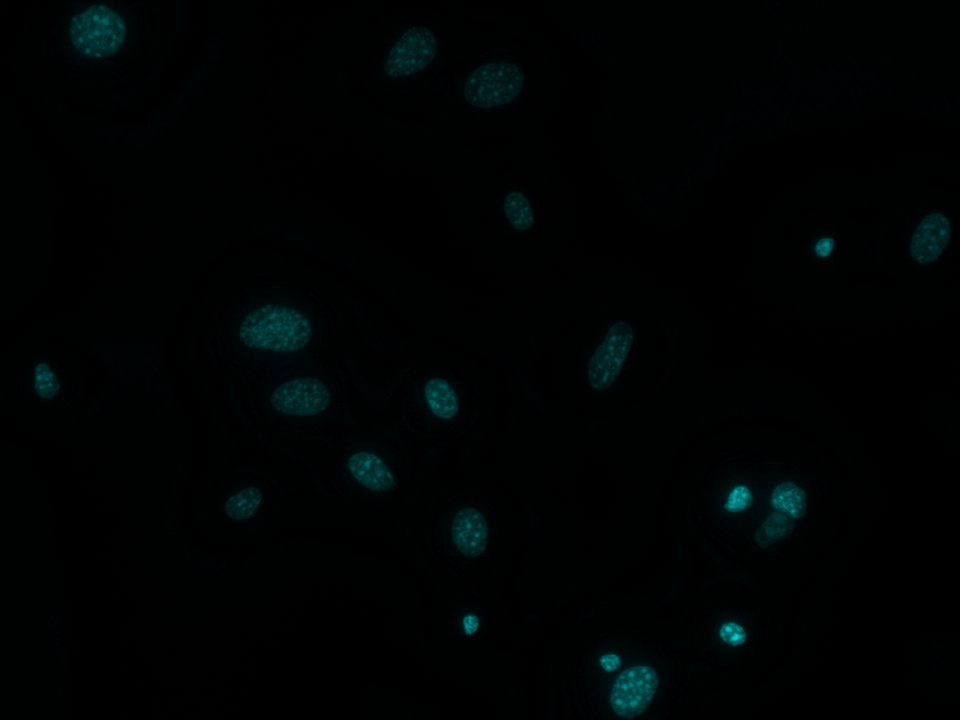

Supplement: Figure 5—figure supplement 1—source data 3. [file elife-91611-fig5-figsupp1-data3.zip › Figure 5-figure supplement 1-source data 3/Sup5C_ZhangL_WT_DAPI_Raw.tif]

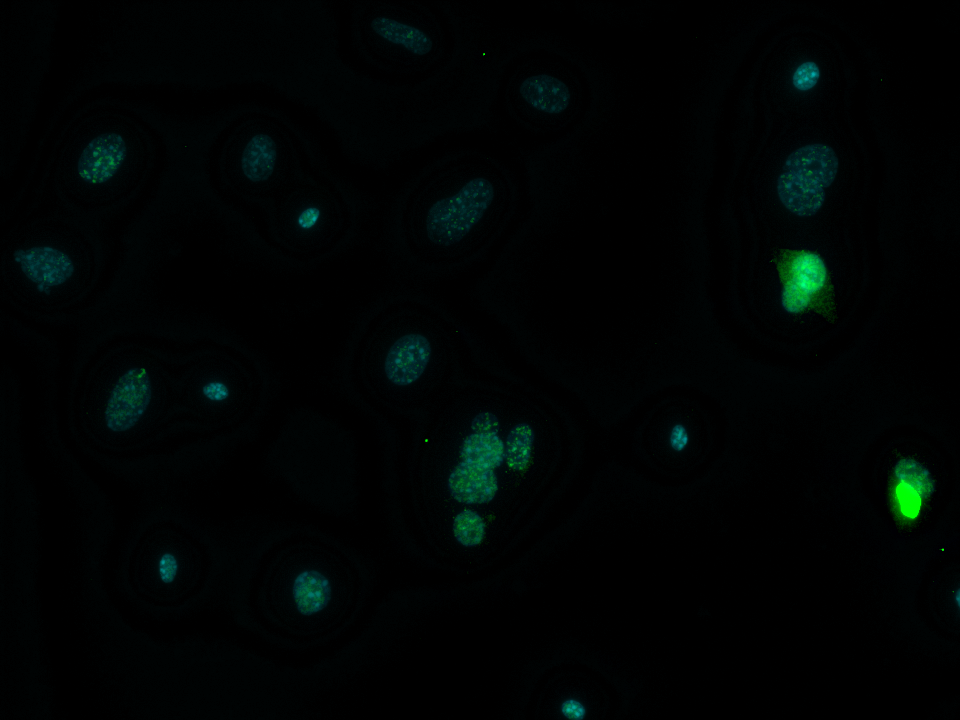

Supplement: Figure 5—figure supplement 1—source data 3. [file elife-91611-fig5-figsupp1-data3.zip › Figure 5-figure supplement 1-source data 3/Sup5C_ZhangL_Ppm1d_Overlay_Raw.tif]

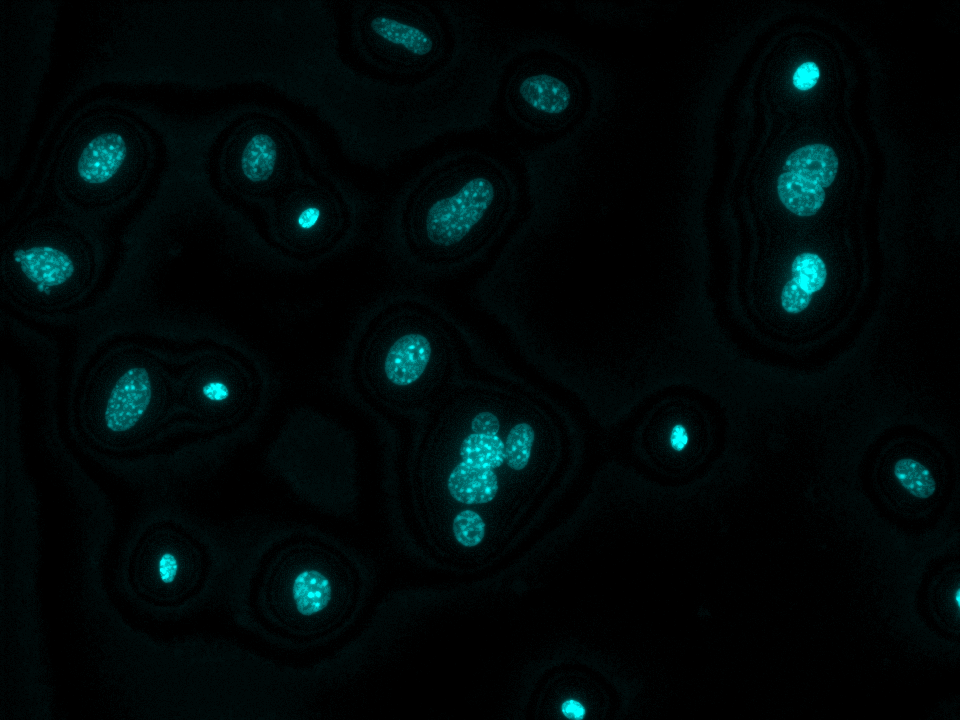

Supplement: Figure 5—figure supplement 1—source data 3. [file elife-91611-fig5-figsupp1-data3.zip › Figure 5-figure supplement 1-source data 3/Sup5C_ZhangL_Ppm1d_DAPI_Raw.tif]

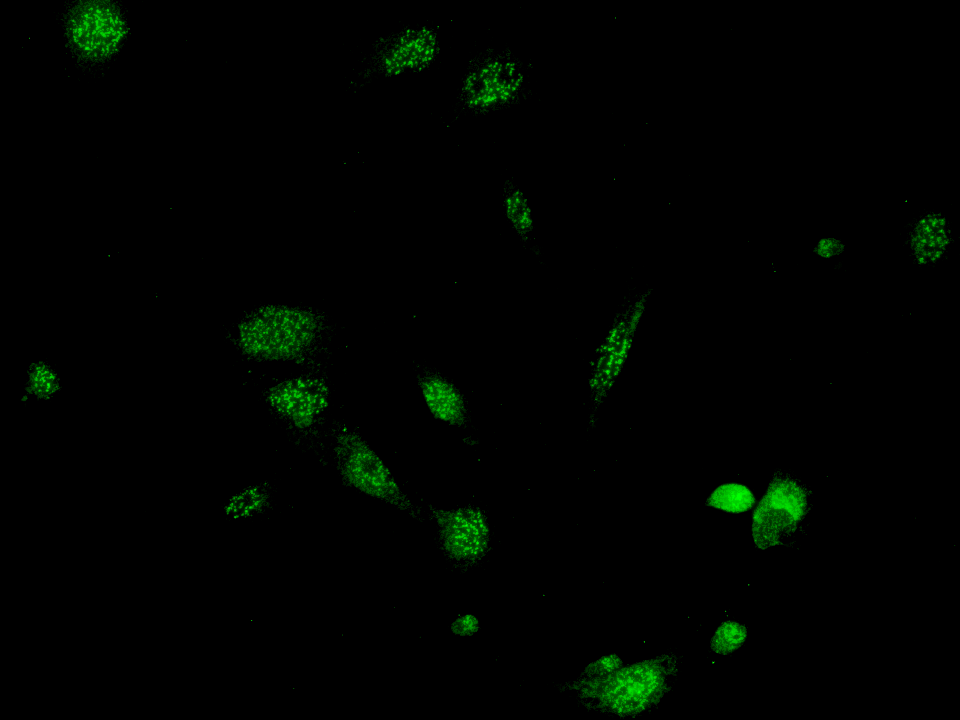

Supplement: Figure 5—figure supplement 1—source data 3. [file elife-91611-fig5-figsupp1-data3.zip › Figure 5-figure supplement 1-source data 3/Sup5C_ZhangL_WT_53BP1_Raw.tif]

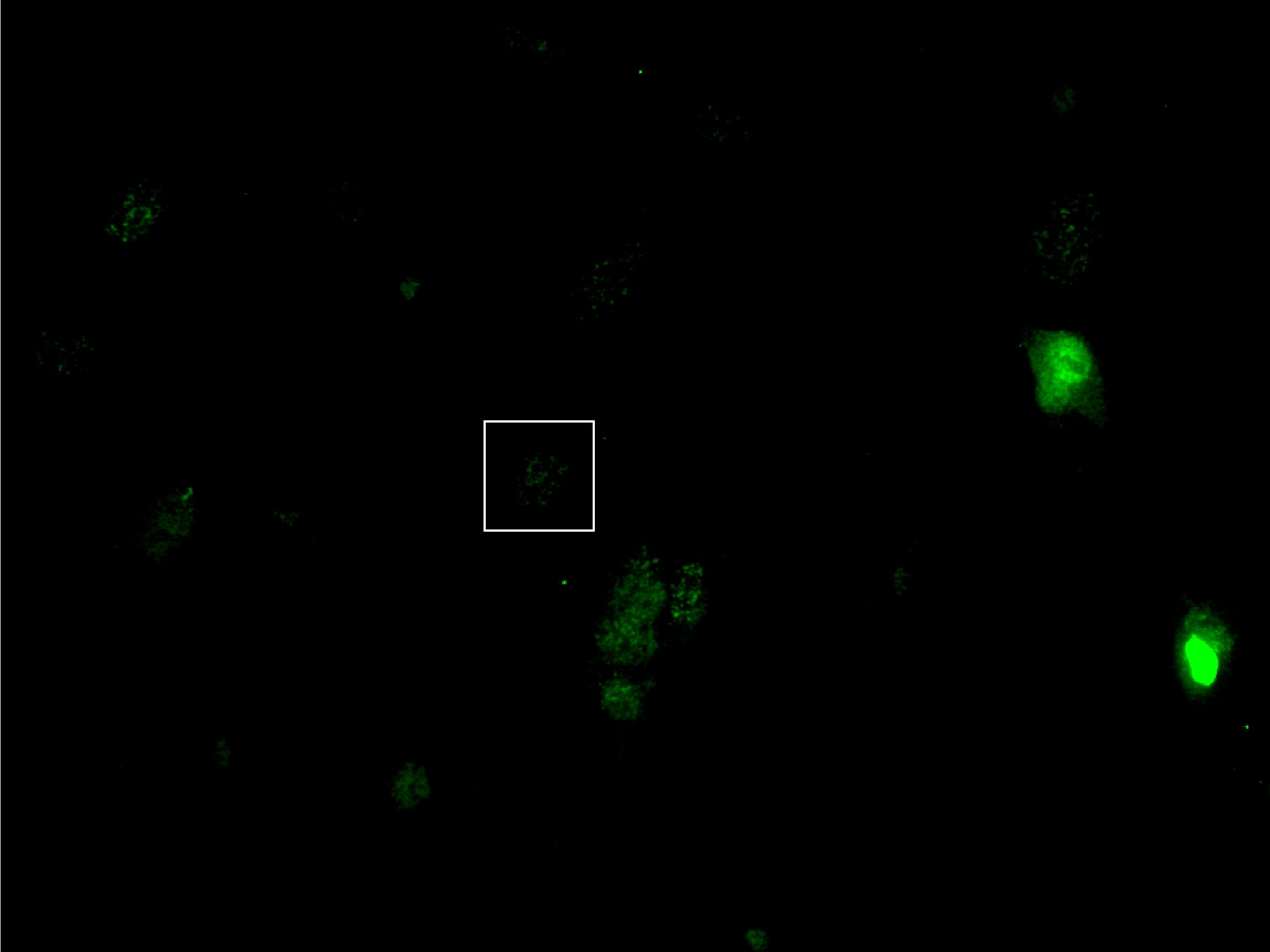

Supplement: Figure 5—figure supplement 1—source data 3. [file elife-91611-fig5-figsupp1-data3.zip › Figure 5-figure supplement 1-source data 3/Supp 5C Annotated Files/Sup5C_ZhangL_Ppm1d_53BP1 An.tif]

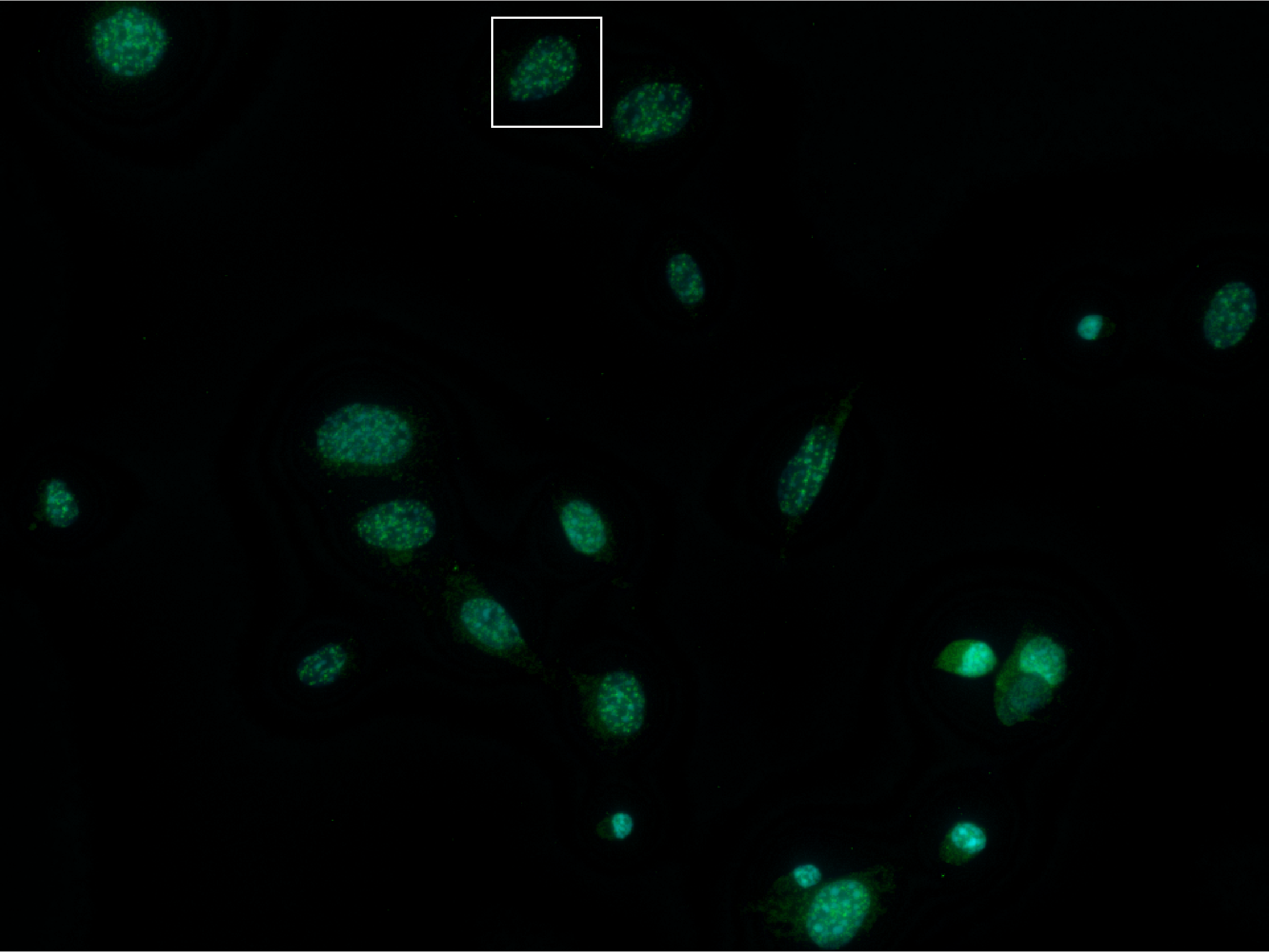

Supplement: Figure 5—figure supplement 1—source data 3. [file elife-91611-fig5-figsupp1-data3.zip › Figure 5-figure supplement 1-source data 3/Supp 5C Annotated Files/Sup5C_ZhangL_WT_Overlay_An.tif]

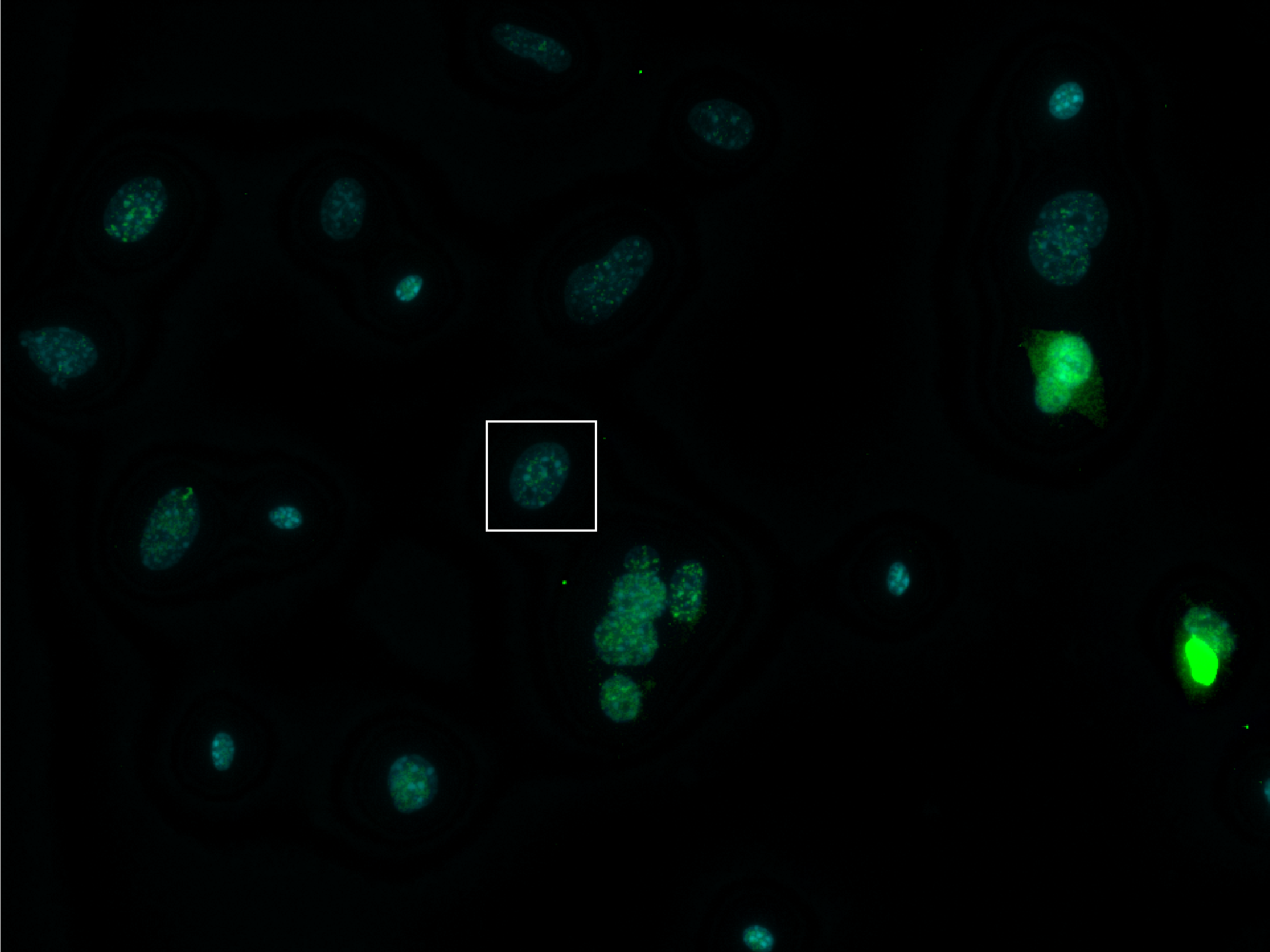

Supplement: Figure 5—figure supplement 1—source data 3. [file elife-91611-fig5-figsupp1-data3.zip › Figure 5-figure supplement 1-source data 3/Supp 5C Annotated Files/Sup5C_ZhangL_Ppm1d_Overlay_An.tif]

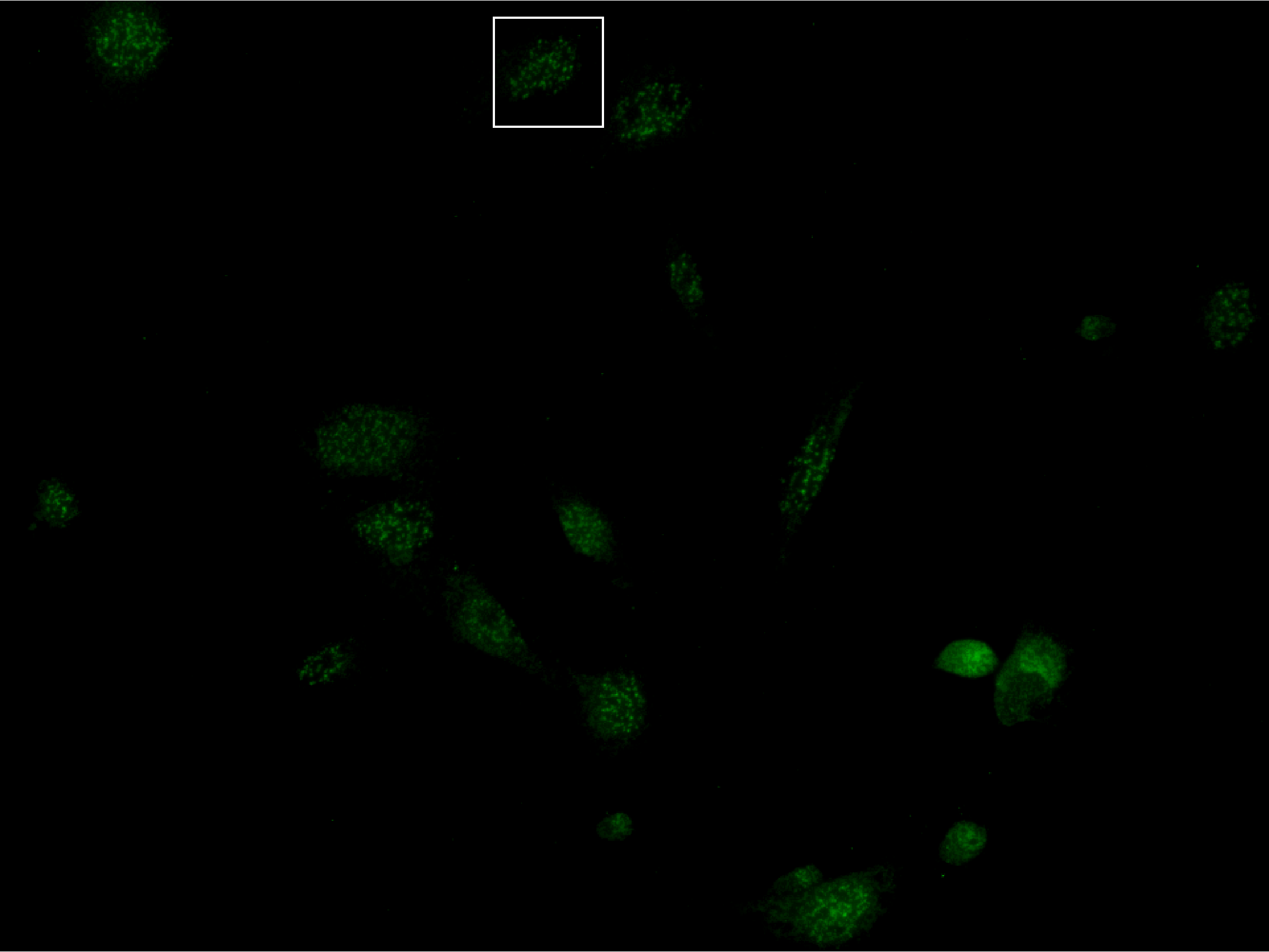

Supplement: Figure 5—figure supplement 1—source data 3. [file elife-91611-fig5-figsupp1-data3.zip › Figure 5-figure supplement 1-source data 3/Supp 5C Annotated Files/Sup5C_ZhangL_WT_53BP1_An.tif]

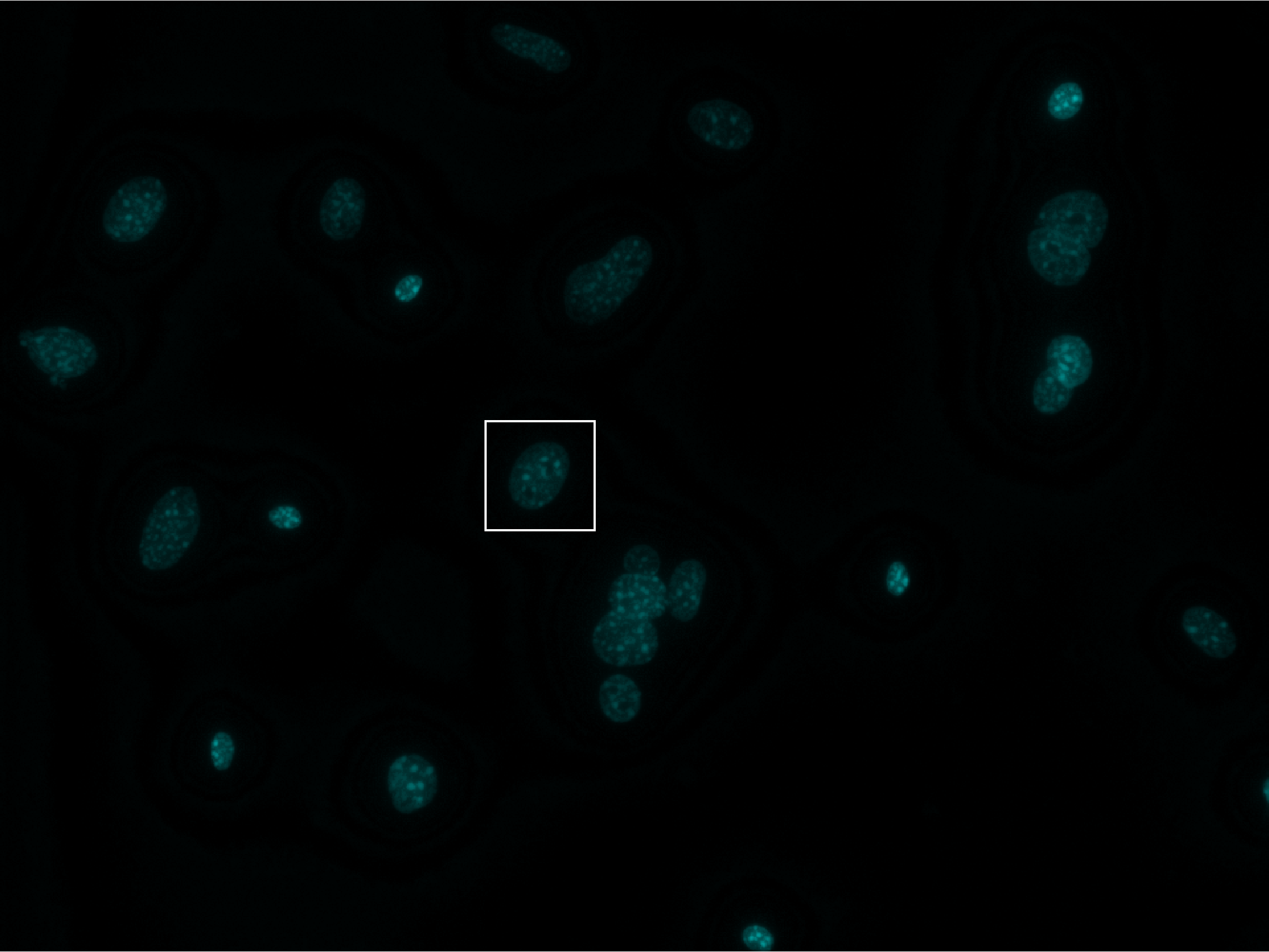

Supplement: Figure 5—figure supplement 1—source data 3. [file elife-91611-fig5-figsupp1-data3.zip › Figure 5-figure supplement 1-source data 3/Supp 5C Annotated Files/Sup5C_ZhangL_Ppm1d_DAPI_An.tif]

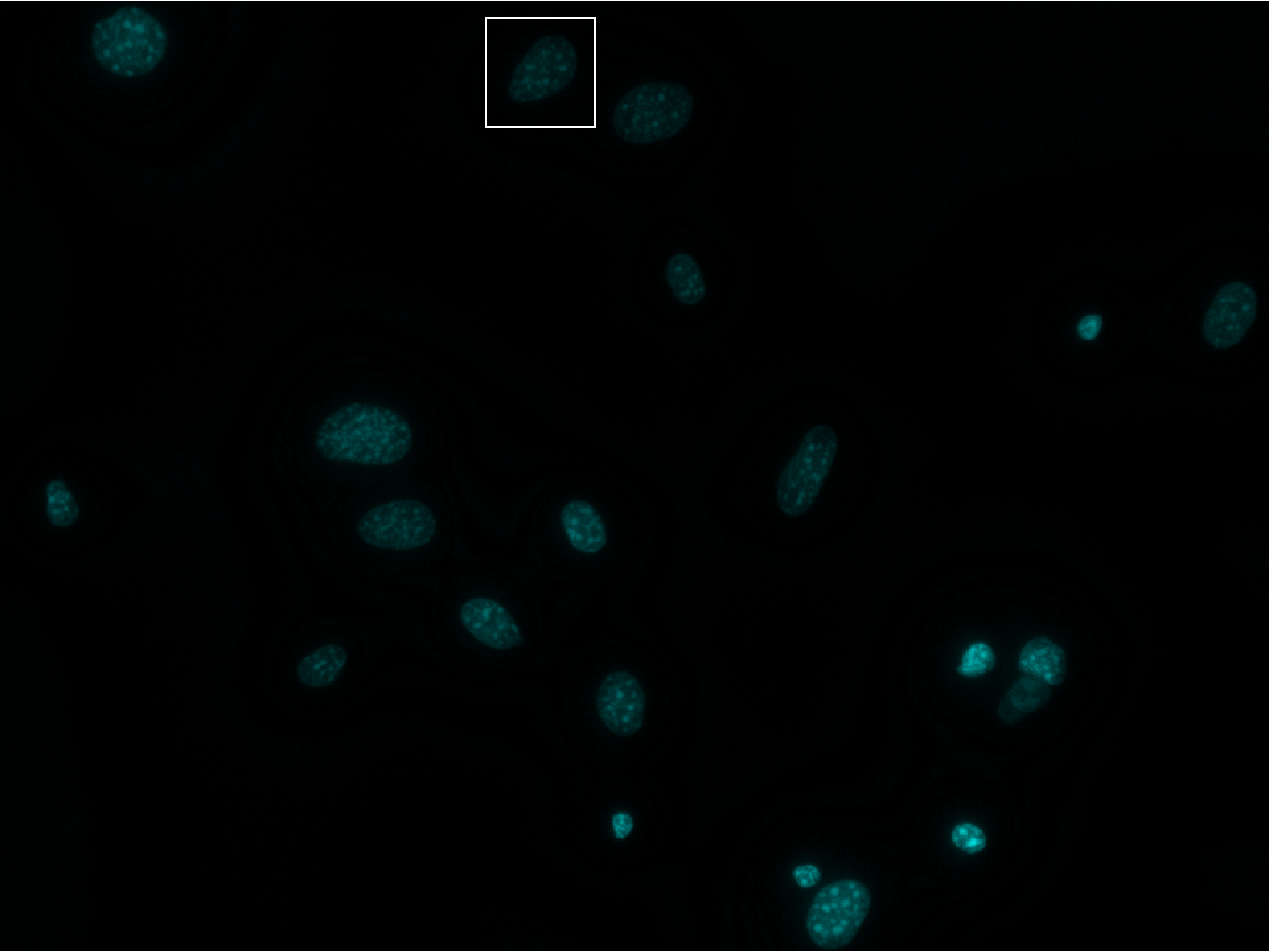

Supplement: Figure 5—figure supplement 1—source data 3. [file elife-91611-fig5-figsupp1-data3.zip › Figure 5-figure supplement 1-source data 3/Supp 5C Annotated Files/Sup5C_ZhangL_WT_DAPI_An.tif]
